# Altered neural anticipation of reward and loss but not receipt in adolescents with obsessive-compulsive disorder

**DOI:** 10.1186/s12888-024-05808-x

**Published:** 2024-05-14

**Authors:** Maria McDonald, Gregor Kohls, Nathalie Henke, Hannes Wahl, Lea L. Backhausen, Veit Roessner, Judith Buse

**Affiliations:** 1 Department of Child and Adolescent Psychiatry, Faculty of Medicine, TUD Dresden University of Technology, Fetscherstraße 74, Dresden, 01307 Germany; 2 Institute of Neuroradiology, Faculty of Medicine, TUD Dresden University of Technology, Dresden, Germany; 3 Clinical Child and Adolescent Psychology, Institute of Clinical Psychology and Psychotherapy, TUD Dresden University of Technology, Dresden, Germany

**Keywords:** Obsessive-compulsive disorder, Adolescence, fMRI, Reward, Loss, Ventral striatum, Orbitofrontal cortex

## Abstract

**Background:**

Obsessive-compulsive disorder (OCD) is characterized by persistent, unwanted thoughts and repetitive actions. Such repetitive thoughts and/or behaviors may be reinforced either by reducing anxiety or by avoiding a potential threat or harm, and thus may be rewarding to the individual. The possible involvement of the reward system in the symptomatology of OCD is supported by studies showing altered reward processing in reward-related regions, such as the ventral striatum (VS) and the orbitofrontal cortex (OFC), in adults with OCD. However, it is not clear whether this also applies to adolescents with OCD.

**Methods:**

Using functional magnetic resonance imaging, two sessions were conducted focusing on the anticipation and receipt of monetary reward (1) or loss (2), each contrasted to a verbal (control) condition. In each session, adolescents with OCD (*n1*=31/*n2*=26) were compared with typically developing (TD) controls (*n1*=33/ *n2*=31), all aged 10-19 years, during the anticipation and feedback phase of an adapted Monetary Incentive Delay task.

**Results:**

Data revealed a hyperactivation of the VS, but not the OFC, when anticipating both monetary reward and loss in the OCD compared to the TD group.

**Conclusions:**

These findings suggest that aberrant neural reward and loss processing in OCD is associated with greater motivation to gain or maintain a reward but not with the actual receipt. The greater degree of reward ‘wanting’ may contribute to adolescents with OCD repeating certain actions more and more frequently, which then become habits (i.e., OCD symptomatology).

**Supplementary Information:**

The online version contains supplementary material available at 10.1186/s12888-024-05808-x.

## Background

Obsessive-compulsive disorder (OCD) is characterized by unwanted thoughts or images that are perceived as frightening, disgusting or shameful (i.e., obsessions), and subsequent or repetitive behaviors (i.e., compulsions). These behaviors often follow strict, self-imposed rules, occur in a stereotypical manner, and are assumed to reduce anxiety or to avoid events or situations that are perceived as frightening or harmful [1]. Accompanied by intrusive thoughts, these repetitive behaviors lead to functional impairment [2]. OCD has two defined subtypes: An early-onset subtype (mean age 11 years, range 9-14 years, defined by starting before age 18) and a late-onset subtype (mean age 23 years) . The early-onset subtype has a prevalence of 1-3 % among in children and adolescents, with high rates of chronicity [3], as approximately 40% of early-onset cases persist into adulthood [4].

Several theoretical accounts exist that attempt to link actual OCD symptoms to neuroimaging findings of reward processing, such that compulsions (like washing or checking) may result from an absent reward signal that normally occurs after completing such tasks [5]. Another facet of OCD suggestive of a possible involvement of the reward system includes the avoidance of potential punishment (i.e., harm avoidance) which is often observed in individuals with OCD (e.g. repetitive, ritualized hand washing to avoid a possible infection of a loved one). Such an attempt to ‘neutralize’ anxiety [2] may indeed be rewarding for the OCD patient, as findings indicate that a significant number of OCD patients report rewarding feelings after completing compulsive behaviors [6]. In addition to harm avoidance or neutralizing anxiety, the behavior of OCD patients may also indicate that they are more sensitive to loss or punishment, which has also been linked to the (dorsal) striatum, including the caudate nucleus in studies conducted in adults with OCD [7, 8]. In fact, aberrant reward system function in OCD has been found in numerous studies using different experimental paradigms [2, 9–12]. Such altered reward function may be of particular interest during adolescence, which is considered a sensitive period with heightened reward responsivity as seen in control populations [13]. However, there are very few studies conducted with adolescents with OCD focusing on reward and loss processing. Interestingly, a recent systematic review of neurocognitive functioning in pediatric patients with OCD (including children and adolescents with this disorder) examined, among other aspects, decision-making based on potential reward gains or losses [14]. This line of research has primarily used the Iowa Gambling Task (IGT), which requires participants to learn over time to select decks of cards that are favorable rather than unfavorable (i.e., they offer a smaller reward but are also less risky to lose). The review concluded that young patients with OCD exhibit impaired decision-making ability by avoiding choices when the outcome is uncertain but likely favorable, which may reflect a desire to be as certain as possible before making decisions. However, because reward and punishment (i.e., losses) are presented intermixed within the IGT, it is not possible to clearly distinguish whether OCD patients’ decisions are due to atypical processing of reward, punishment, or both.

Neurally, OCD has been consistently associated with alterations in the cortico-striatal-thalamo-cortical (CSTC) circuits [15]. More recent research has further refined these circuits and linked neural alterations to patients’ reports of their OCD symptoms. Although not without limitations, this has resulted in verifiable clinical profiles underscored by neurocognitive alterations in five distinct neurocircuits [15]. For the current study, the ventral-affective circuit that is mainly comprised of orbitofrontal cortex (OFC), thalamus and basal ganglia, especially the ventral striatum (VS, incl. nucleus accumbens) [16, 17] is of interest. Regions of this circuit have been linked to reward-related processing, including reward ‘wanting’ (VS) and reward ‘liking’ (OFC), and seem to be altered in OCD [2, 18]. In particular, the VS appears to be important in the pathophysiology of OCD, as this region is one of the main targets of deep brain stimulation for the treatment of treatment-refractory OCD patients [19, 20]. Studying these regions in a young sample of OCD patients who are just after the onset of their disorder and hence not as affected by many years of illness as adult patients with OCD is of great interest to better understand the disorder.

One of the most commonly used tasks that reliably recruits the VS and OFC during reward and loss processing in fMRI is the Monetary Incentive Delay (MID) task [21, 22]. In this task, the blood-oxygen level dependent (BOLD) response of an experimental condition (e.g., monetary condition) is compared to a control condition (e.g., verbal condition) each signaled by a different condition cue at the beginning of a trial. Then a target stimulus appears to which the participants should react as quickly as possible by pressing a button, followed by feedback on their performance. Two distinct phases of reward and loss processing can be examined separately within this task – anticipation and receipt [23].

Typically, faster reaction times are observed for the monetary condition compared to the control condition in the MID task, suggestive of higher motivation to gain a reward or not lose money [2]. Using this task, previous research revealed mixed behavioral findings for anticipating monetary rewards in adult patients with OCD versus typical controls ranging from no reaction time (RT) differences [18] to slower RTs [2]. Neuroimaging studies have also demonstrated inconsistent findings ranging from no differences to decreased but also increased VS and OFC activity in adults with OCD compared to typical controls which might be linked to differences in task design or symptom severity. Although 76% of OCD cases are classified as early-onset [24] there are no reward studies using the MID task conducted in adolescents with OCD to date. Thus, a thorough investigation of reward system functioning is needed to gain more insight into the neural mechanisms underlying OCD-related behavior, particularly in affected youth.

Based on various theoretical models of OCD (e.g., [25]), the current fMRI study aimed to investigate whether brain activation in the VS and OFC differs between adolescents with OCD and TD during reward and loss processing. We hypothesized that RTs and the extent of brain activation in the two regions-of-interest (ROI), VS and OFC, would differ between the OCD and TD groups during the anticipation phase in a MID task for reward and loss. Given the mixed results in the adult literature, we refrained from specifying the direction of the expected divergent brain activation. We also hypothesized that the ROI activation of adolescents with OCD would differ from that of the TD group during the feedback phase for both rewards and losses. Finally, we assumed an association between OCD symptom severity and VS as well as OFC activation within the OCD group during both sessions, but did not make a directional prediction due to the inconsistent findings in the available literature.

## Methods

### Participants

Seventy-seven adolescents with obsessive-compulsive (OC) symptoms and 44 TD adolescents aged between 10-19 years were recruited for a larger research project through online advertisement, inpatient treatment, the local databank of the Department of Child and Adolescent Psychiatry at TU Dresden as well as through psychotherapists in private practice. This work was part of a larger study focusing on reward and loss processing as well as the influence of mindfulness apps on emotion regulation and cognitive control in OCD. Data from control participants in the current study were also used in two previously published studies dealing with reward and loss processing comparing typically developing adolescents and healthy adults [26, 27]. The current study is substantively different as it focuses on a clinical population: adolescents with OCD. Before participating, a telephone screening to check for general exclusion criteria (e.g., MRI contraindications, color blindness) took place (for diagnoses and medication status of the sample see S1). Afterwards, in a first assessment appointment, all participants were screened for lifetime psychiatric disorders using the Mini-International Neuropsychiatric Interview (MINI/MINI Kid [28]). Of the initially contacted 77 adolescents with OC symptoms, 22 were excluded due to other prominent diagnoses (e.g., ADHD, tic disorders) or OC symptoms below CY-BOCS values of 8 which were predefined as cut-off. Further, 12 were excluded due to MRI contraindication, one due to left-handedness as measured by the Edinburgh Handedness Inventory [29] and 3 dropped out from study participation. In addition, 3 TD were excluded due to current and/or past mental health problems according to the MINI Kid [28], and 1 dropped out before the initial assessment leaving 39 adolescents with OCD and 40 TD eligible for the scanning sessions. Before the scanning, 4 adolescents with OCD and 1 TD decided against taking part in the scanning session. Diagnoses in the OCD group were given using ICD-10 criteria [30] by a board certified child and adolescent psychiatrist. Adolescents with OC symptoms were interviewed using the Children’s Yale-Brown Obsessive-Compulsive Scale (CY-BOCS [31, 32]; see Tables 1 and 2 for sample characteristics). After scanning, exclusion of participants was due to excessive head movement (> 3mm; reward session *n*=1 OCD; loss session *n*=5 OCD), too many error trials (i.e., more than 33% errors per condition; reward session *n*=2 TD; loss session *n*=1 OCD), substantial anatomical abnormalities (e.g., too large ventricles; reward session *n*=1 OCD, *n*=2 TD; loss session *n*= 1TD), experimental error or technical problems (reward session *n*= 1 TD; loss session *n*=1 OCD, *n*=1TD), later OCD diagnosis in TD (reward session *n*=1 TD; loss session *n*=1 TD) and other reasons (reward session *n*=2 OCD; loss session *n*=2 OCD, *n*=5 TD). The final analysis sample for the reward session consisted of 31 OCD and 33 TD and the loss session of 26 OCD and 31 TD (note that out of all participants 25 OCD and 28 TD participated in both sessions). Participants completed two separate, counterbalanced MRI sessions at least 14 days apart. Note that the majority of the participants were of white (i.e., Caucasian) European ancestry. Still, detailed information on race and/or ethnicity was not systematically collected in accordance with ethical guidelines in Germany.

**Table 1:** Demographics and clinical characteristics (*n* *= 64*) for the reward session.

	*OCD (n = 31)*		*TD (n = 33)*		*Comparison between groups*			95% CI
	*M (SD)*	*range*	*M (SD)*	*range*	*t/chi*^*2*^	*df*	*p*	
Age in years	15.48 *(2.03)*	11.11 – 19.07	15.55 *(1.91)*	10.46 – 18.90	-0.14	62	0.89	[-1.05 – 0.91]
% of females	61.3 %		54.5 %		0.30	1	0.59	
Current medication, n	13							
Socioeconomic status^a^	14.29 (*3.70*)	7.00 – 21.00	16.33 (*3.51*)	10.00 – 23.00	-2.27	62	0.03	[-3.84 – -0.24]
Perceptual speed^b^	106.24 (*15.22*)	86.50 – 139.00	109.91 (*13.99*)	86.50 – 143.50	-1.00	62	0.32	[-10.97 – 3.63]
Pubertal status^c^	3.74 *(.86)*	2.00 – 5.00	3.79 *(.82)*	2.00 – 5.00	-0.22	62	0.83	[-0.47– 0.37]
Money received in €	26.82 (*1.73*)	22.00 – 29.50	25.97 (*1.97*)	22.00 - 29.50	1.83	62	0.07	[0.08 – 1.78]
**OCI-R**^d^	22.19 (15.79)	4.00 – 59.00	6.52 *(5.91)*	0.00 – 24.00	5.20	37.80	< 0.001	[9.57 –21.78]
**ZWIK – Self**^e^ Total score	40.26 *(30.68)*	2.00 – 128.00	8.94 *(6.97)*	0– 23.00	5.55	32.91	< 0.001	[19.84 – 42.80]
**ZWIK – Parents**^e^ Total score	37.13 *(23.03)*	5.00 – 103.00	2.42 (*2.91*)	0– 10.00	8.33	30.90	< 0.001	[26.21 – 43.20]
**CY-BOCS**^f^								
Obsessions	7.26 *(3.90)*	0 – 15.00						
Compulsions	7.77 (3.03)	3.00 – 14.00						
Total score	15.03 *(5.97)*	8.00 – 28.00						

^a^Following Winkler & Stolzenberg (2009) the calculation of the socioeconomic status contained parents’ school and professional education, recent professional status and family income. A family-based measure of socioeconomic background was achieved by averaging scores of mothers and fathers ranging from 3 to 21. Higher values indicate a higher socioeconomic status

^b^Processing speed was estimated using the Zahlen-Verbindungs-Test [33]

^c^Pubertal developmental Scale [34]

^d^Obsessive-Compulsive Inventory-Revised [35]

^e^Zwangsinventar für Kinder und Jugendliche [36]

^f^Children’s Yale-Brown Obsessive-Compulsive Scale (CY-BOCS; Goodman et al., 1991; Steinhausen, 2007) which total severity score ranges from 0 to 40.Values between 8 and 15 are considered mild, 16-23 moderate, 25-30 severe and over 30 extreme [37]

**Table 2:** Demographics and clinical characteristics (*n* = 57) for the loss session

	*OCD (n = 26)*		*TD (n = 31)*		*Comparison between groups*			*95% CI*
	*M (SD)*	*range*	*M (SD)*	*range*	*t/chi*^*2*^	*df*	*p*	
Age in years	*15.48 (1.92)*	*11.11 – 18.90*	*15.62 (1.93)*	*11.31 – 18.90*	*-0.32*	*55*	*0.75*	*[-1.19 – 0.86]*
% of females	*61.5 %*		*51.6 %*		*0.57*	*1*	*0.45*	
Current Medication, n	*11*							
Socioeconomic status^a^	*13.96 (3.53)*	*7.00 – 21.00*	*17.00 (3.40)*	*10.0 – 23.00*	*-3.20*	*55*	*0.002*	*[-4.88 – -1.20]*
Perceptual speed^b^	*105.83 (14.98)*	*86.50 – 139.00*	*110.79 (14.57)*	*86.50 – 143.50*	*-1.27*	*55*	*0.21*	*[-12.83 – 2.90]*
Pubertal status^c^	*3.69 (0.79)*	*2.00 – 5.00*	*3.84 (0.90)*	*0.00 – 5.00*	*-0.65*	*55*	*0.52*	*[-0.60 – 0.31]*
Money received in €	*16.46 (1.77)*	*12.50 – 19.00*	*15.90 (1.72)*	*11.50 – 18.00*	*1.20*	*55*	*0.23*	*[0.37 – 1.49]*
**OCI-R**^d^	*20.08 (14.72)*	*4.00 – 59.00*	*7.42 (6.90)*	*0.00 – 24.00*	*4.03*	*34.11*	*< 0.001*	*[6.27 – 19.04]*
**ZWIK – Self**^e^ Total score	*35.85 (28.77)*	*2.00 – 128.00*	*9.90 (8.12)*	*0.00 – 30.00*	*4.45*	*28.35*	*< 0.001*	*[14.01 – 37.88]*
**ZWIK – Parents**^e^ Total score	*37.12 (25.39)*	*5.00 – 103.00*	*2.80 (3.26)*	*0.00 – 10.00*	*7.45*	*52*	*< 0.001*	*[24.69 – 45.00]*
**CY-BOCS**^f^								
Obsessions	*7.769 (4.59)*	*0 – 16.00*						
Compulsions	*8.423 (3.52)*	*3.00 – 15.00*						
Total score	*16.192 (7.24)*	*8.00 – 31.00*						

^a^Following Winkler & Stolzenberg (2009) the calculation of the socioeconomic status contained parents’ school and professional education, recent professional status and family income. A family-based measure of socioeconomic background was achieved by averaging scores of mothers and fathers ranging from 3 to 21. Higher values indicate a higher socioeconomic status

^b^Processing speed was estimated using the Zahlen-Verbindungs-Test [33]

^c^Pubertal developmental Scale [34]

^d^Obsessive-Compulsive Inventory-Revised [35]

^e^Zwangsinventar für Kinder und Jugendliche [36]

^f^Children’s Yale-Brown Obsessive-Compulsive Scale (CY-BOCS [31, 32]) which total severity score ranges from 0 to 40.Values between 8 and 15 are considered mild, 16-23 moderate, 25-30 severe and over 30 extreme [37]

### Experimental paradigm, design, and procedure

**Fig. 1:**
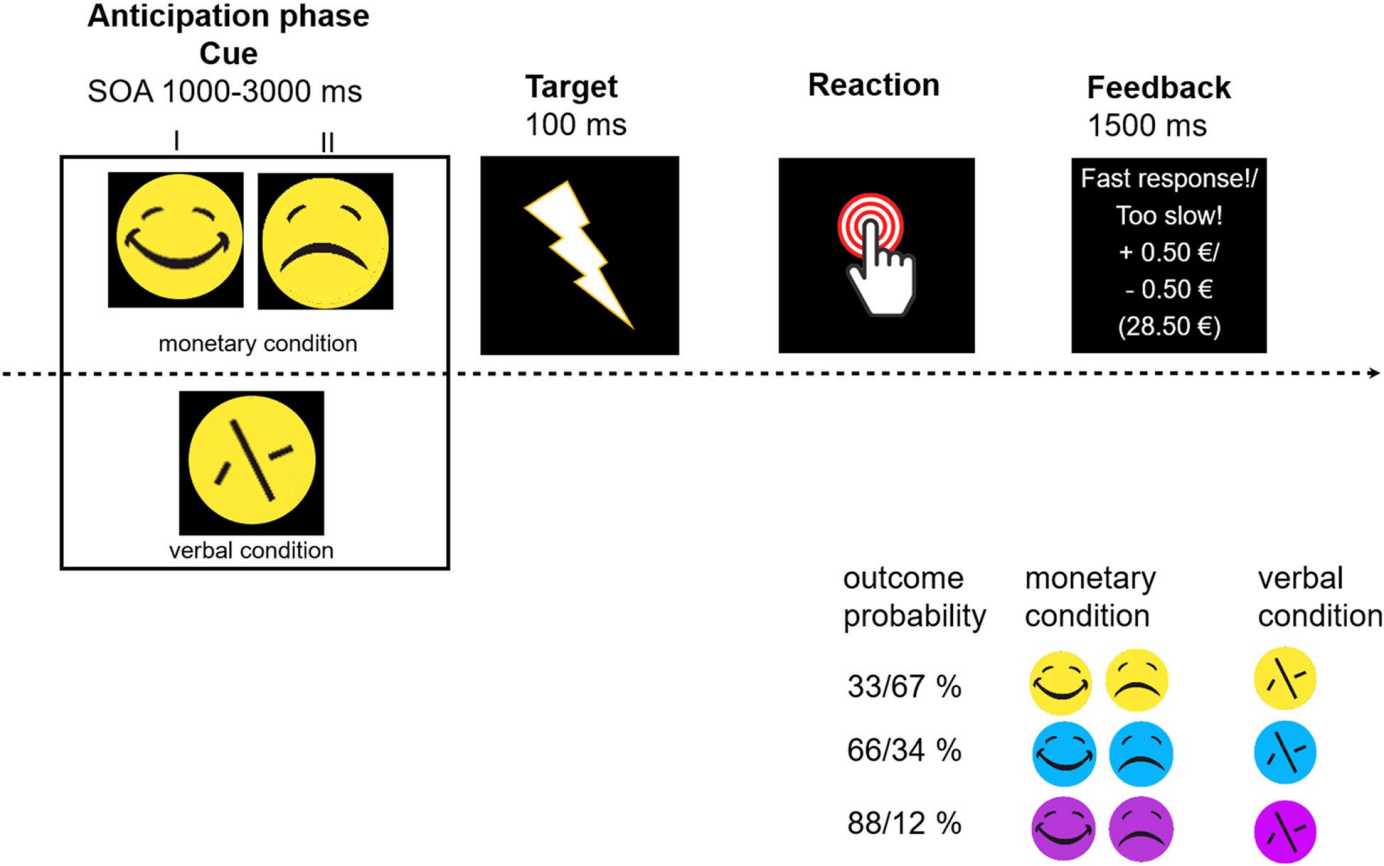
Example trial of the MID task including three different outcome probabilities. Session I – Reward: During the anticipation phase, participants were presented one of two cues, either a happy face (monetary condition) or a scrambled face (verbal condition, i.e., control condition) to anticipate a potential reward or neutral trial. Session II – Loss: Participants were presented either a sad face (monetary condition) or a scrambled face (verbal control condition). The cue indicated a monetary or verbal control trial. Cue presentation served as the anticipation phase in the MID task. During the reward session, the color yellow symbolized the 33%, blue 66%, and pink 88% outcome probability. During the loss session, yellow symbolized the 67%, blue 34%, and pink the 12% outcome probability. This was an implicit condition, participants therefore were not aware of this association between color and outcome probability. During both sessions, participants were instructed to react as fast as possible towards a flash (target) that appeared after the cue. The flash was realized by the black screen turning white for 100ms. The time between cue and target varied (1000-3000 ms). Each trial concluded with a feedback regarding the reaction time. Feedback presentation was the feedback phase of the task. Depending on the session, they only gained or did not lose money if they reacted within the valid RT window for the respective outcome probability. The paradigm constantly adapted to the individual reaction time of the participant. Participants were not aware which color represented which outcome probability. SOA= Stimulus Onset Asynchrony; ITI = Intertrial Interval 2000 ms

A modified version of the Monetary Incentive Delay (MID) task [21, 26, 27] was utilized to examine behavioral and neural activity during the anticipation and feedback phase of reward and loss. The task was adapted to examine reward and loss processing as a function of different outcome probabilities each represented by differently colored cue stimuli (see Fig. 1).

Ninety trials were presented per condition (i.e., money, and verbal in which only verbal feedback was provided on trial performance; 30 trials for each outcome probability) and a total of 180 trials for each session (i.e., reward and loss). Outcome probabilities were linked to differences in task difficulty as the valid RT window for hitting the response button on time varied and adapted to the individual RT of the participants over the course of the task (see for detailed calculation of the outcome probabilities S2). Each trial started with a cue stimulus – a monetary (indicated by a happy or sad smiley, depending on the session) or a verbal cue (indicated by a scrambled smiley) - presented on a black screen. This was followed by a flash (Stimulus Onset Asynchrony (SOA) = 1000 – 3000 ms, mean 2000 ms) achieved by the black screen turning white for 100ms. Participants were instructed to react as fast as possible after the flash by using the right index finger. During the reward session, participants gained 0.50 € in the monetary condition or received a verbal feedback (i.e., verbal condition) with the virtual deposit being unchanged when reacting within a valid RT window. The procedure was similar in the loss session. However, in this session, participants started with a virtual deposit of 35€ and lost 0.50€ if they did not react within the valid RT window during the monetary condition. An example trial of the paradigm is depicted in Fig. 1. We are aware that this task design incorporates a possible confound between outcome probability and task difficulty (i.e., trials get more difficult to win/avoid losing with lower outcome probability as the valid reaction time window is shorter compared to the higher outcome probabilities; see S2). As a result, cognitive effort in this paradigm is conceptualized through increasing task difficulty. This task design allows to distinguish between a more motivational response (i.e., increasing VS activation with increasing probability) and cognitive effort (i.e., highest VS activation for lowest probability). It thus offers a new addition to previous MID task designs and could help to better understand the role of the VS for reward/loss processing in OCD. It should be noted, though, that the effect of cognitive effort could be asserted either because participants think it is easy (i.e., slower reaction times necessary for reaching the valid reaction time window) or because the probability of gaining a reward/avoiding a loss is high; this cannot be clearly distinguished in this paradigm.

Task presentation was controlled using Presentation® software (Version 20.1 Build 12.04.17, Neurobehavioral Systems, Inc., Albany, CA) and button presses were recorded with ResponseGrips (©NordicNeuroLab). Prior to the scanning, participants did a practice session outside the scanner consisting of 20 trials to familiarize themselves with the task in which cue color and outcome probability were linked the same way as in the actual scanning session. Participants could further ask questions, but they did not receive any money during this practice session. But participants saw a box of cash before scanning to induce motivation.

Details regarding the amount of money that each participant received can be found in Tables 1 (reward) and 2 (loss). The scanning time of the paradigm and the structural T1 took approximately 35 minutes per session. The session concluded with a rating outside the scanner in which the cue stimuli were presented again. Participants were asked to rank the anticipated reward (or loss) from 1 (lowest reward or loss) to 3 (highest reward or loss). Both groups did not differ regarding their ratings (see S3 for details).

### Functional brain imaging

#### Image acquisition

A 3-T whole body scanner (MAGNETOM Prisma, Siemens, Erlangen, Germany) was equipped with a 64 channel head coil to acquire the (f)MRI scans. T2* weighted single shot gradient echo planar imaging (EPI) sequences were used to obtain fMRI data (TR/TE = 3000/30ms, 2 × 2 × 2 mm voxel size, FOV 192 x 192mm, 49 axial slices, flip angle = 90°). Data were real-time motion corrected using prospective acquisition correction (PACE) technique. The first five EPI volumes were discarded to allow for T1 equilibration. The paradigm was presented on a MR-suitable LC-Display located behind the scanner bore. A mirror was mounted on the head coil for participants to see the paradigm. After the acquisition of the functional data, a high resolution 3D T1-weighted magnetization-prepared rapid gradient echo (MPRAGE) was acquired with the following parameters: TR/TE = 2400/2.23 ms, FOV = 272 × 272 mm, 240 sagittal slices, 0.85 × 0.85 × 0.85 mm isotropic voxel size, flip angle = 8°. These parameters ensured a whole brain coverage only omitting the inferior part of the cerebellum. Structural data were screened for anatomical anomalies and motion artifacts.

#### Analysis of RTs

Statistical analyses were performed using SPSS (IBM SPSS Statistics for Windows, Version 28.0). Overall mean RTs and standard deviations were calculated for each participant and only included within a range of mean ± 2 *SD* to exclude outliers as done in previous studies [26, 27]. In addition, we excluded errors from the analysis. Errors were defined as too early responses (RT < 100ms) or if the participant did not react towards the flash (see S4 for details). RTs were analyzed using a 2 x 2 x 3 ANOVA with group (OCD versus TD) as a between-subject-factor and condition (monetary versus verbal condition) and outcome probability (33%/67% vs. 66%/34% vs. 88%/12%) as within-subject-factors for each session separately. We chose to analyze the two sessions separately because the data were also collected in two separate scanner sessions on two separate days. In addition, the verbal condition differs in whether it occurred alongside possible rewarding or loss trials, so that different influences on the verbal condition cannot be completely ruled out and could have an impact, especially when calculating difference values. When the Mauchly-test indicated violations of sphericity, Greenhouse-Geisser-adjustments were used. Post-hoc tests were Bonferroni-corrected when necessary. We applied a significance threshold of *p* ≤ .05. Partial eta-squared (0.01 small, 0.06 medium and 0.14 large) and Cohen’s d were used as effect size measures [38].

#### Preprocessing of fMRI data

Functional imaging data were analyzed using Statistical Parametric Mapping (SPM12, Wellcome Department of Cognitive Neurology, London, UK) implemented in MATLAB (2019a, Math Works, MA, USA). The following steps were included in the pre-processing pipeline: (1) Slice-time correction with the middle slice as reference, (2) realignment with 6 degrees of freedom to the first volume as well as unwarping for motion correction. If head movement of the participants was greater than 2.5 mm or 2.5 degrees from volume to volume, the whole data set of the participant was excluded (see S1 for details). (3) A rigid coregistration with 6 degrees of freedom of the individual anatomical image to the mean functional image was used. Further pre-processing steps were (4) segmentation of the coregistered anatomical image (without reslicing) to calculate spatial normalization as well as the brain mask needed for the first-level analysis, (5) normalization of the structural images by using a customized template created with the Template-O-Matic (TOM) toolbox [39], and (6) smoothing by using a Gaussian kernel with a full-width half-maximum (FHWM) of 6 mm.

#### Statistical analyses at the whole-brain level

Whole-brain level analyses were conducted within a GLM framework and used the same factors as for the analysis of the behavioral data (see S5 for details regarding the factors of the first-level analyses). Additionally, error trials (no reaction towards the flash or not within an individually calculated range of mean ±2 *SD*s), target, keypress and six movement regressors that resulted from rigid body realignment were included as regressors of no interest within the model. For results please see S9, S10 (reward) and S11, S12 (loss).

#### Region-of-interest (ROI) analyses

Based on previous evidence of VS, and OFC involvement in OCD [2] and in reward and loss processing [40], these regions were selected as ROIs. Percent signal change (PSC) was extracted for each of these regions using rfxplot [41]. Our ROI masks “N_Acc” (as a proxy for the VS) and “OFC” were derived from AAL3 [42] in Wake Forest University (WFU) PickAtlas [43, 44] and coregistered to the generated TOM template used in the study. The OFC mask included all subparts from the WFU pickatlas. Using an age-adjusted tissue probability map created using the TOM8-toolbox as the reference for SPM-segmentation allows the further use of SPM warping maps to normalize adolescent data to the MNI standard space also used by AAL3. Afterwards, the 2x2x3 ANOVA (factors group, condition, and outcome probability) for the anticipation phase was computed using SPSS and included the same factors as used for the analysis of the behavioral data. The 2x2x2 ANOVA for the feedback phase compromised the factors group (OCD vs. TD), condition (monetary vs. verbal condition) and feedback (fast enough vs. too slow). Whenever necessary, post-hoc tests were Bonferroni-corrected. Presentation of results focuses on group differences. As the socioeconomic status (SES) differed between both groups, we further conducted analyses that included SES as a covariate.

#### Correlation of behavior and neural activation in the ROIs and neural activation and severity

To examine possible correlations between severity of obsessions, compulsions and the total score of the CY-BOCS and PSC in the ROIs during anticipation and feedback phase in both sessions, bivariate correlations were calculated for the monetary and verbal condition separately. For statistical hypothesis testing, α was set at 0.05. In order to correct for multiple testing, a Bonferroni correction was applied (α´ = 0.004).

## Results - Session I: Reward

### Behavioral results – Reaction times

The 2x2x3 ANOVA revealed no main effect of group (*F*_(1, 62)_= 3.27, *p*= 0.08, *η*_*p*_^*2*^= *0.05).* A main effect of condition (*F*_(1, 62)_= 77.26, *p* < 0.001, *η*_*p*_^*2*^= 0*.*56) with faster RTs in the monetary compared to the verbal condition (M_monetary_= 244.51± 28.51; M_verbal_= 259.28±33.21) and of outcome probability (*F*_(1.215,124)_= 171.57, *p* < .001, *η*_*p*_^*2*^= 0*.74)* occurred*.* Participants demonstrated fastest RTs at 33% and slowest RTs at 88% outcome probability (M_33%_= 226.95±22.66; M_66%_= 244.73±29.19; M_88%_=262.74±37.51; t_33_66%_(63) = -15.24, *p* < 0.001, d= -1.91; t_33_88%_(63)= -14.03, *p*< 0.001, d= -1.75; t_66_88%_(63)= -9.99, *p* < 0.001, d= -1.25). No further interactions were found (all *ps* > 0.075; for details see S6). Additional analyses with SES as a covariate can be found in S7.

### fMRI results

#### Anticipation phase – region-of-interest (ROI) analysis

**Fig. 2:**
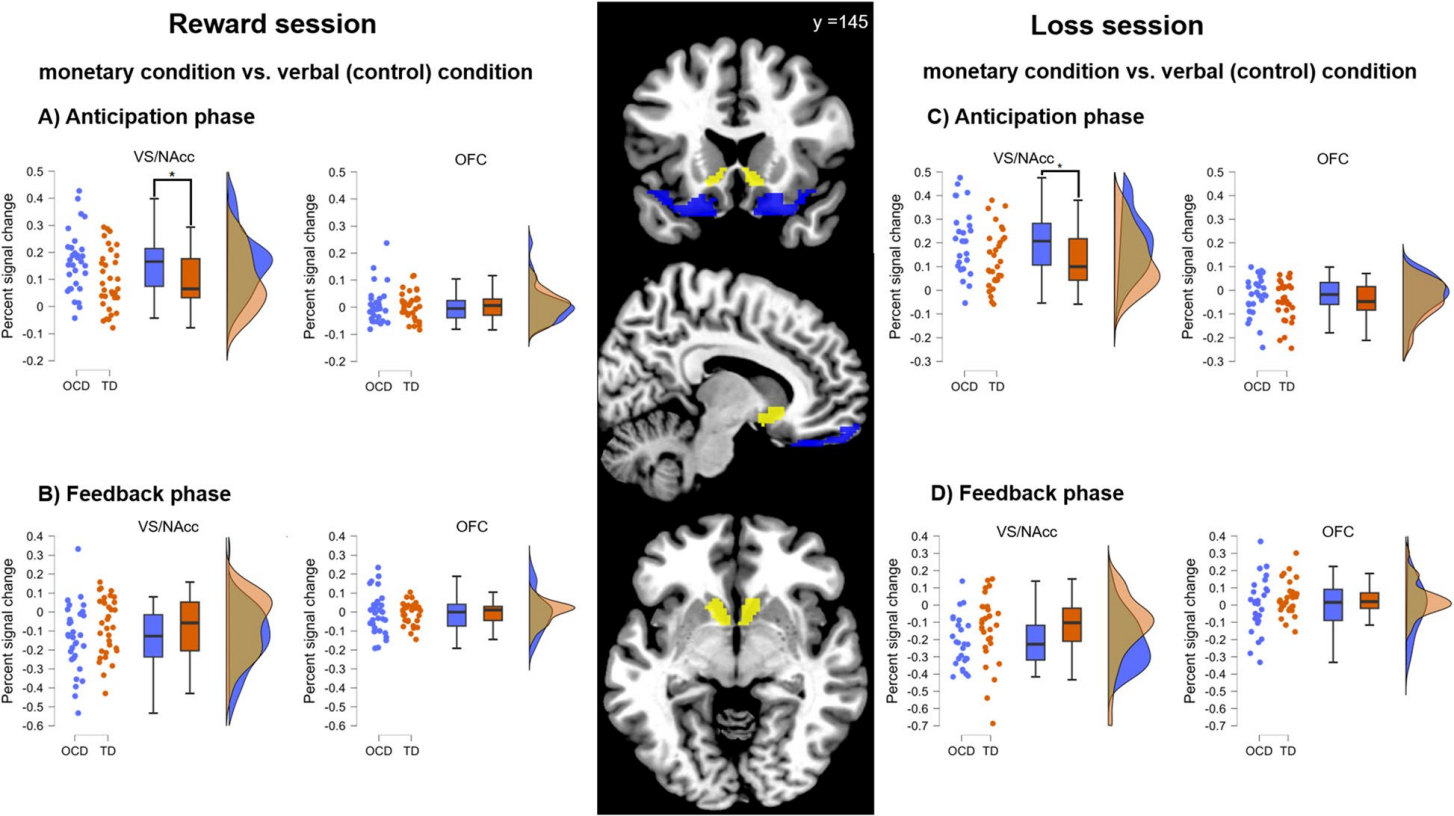
Group comparions of percent signal change in the bilateral ROIs (brain images in the middle: VS= yellow, OFC= blue) during the anticipation and feedback phase of a MID task in the reward and loss sessions. Depicted are difference values between monetary and verbal (control) condition for A) Reward anticipation phase, B) Reward feedback phase, C) Loss anticipation phase and D) Loss feedback phase. The raincloud plots show individual data points, box plots and a distribution plot for each group. The adolescent OCD group is depicted in blue colour, the adolescent TD group in orange. * *p* < 0.05 (Bonferroni-corrected for multiple comparions). Raincloud plots were created using JASP [45]

In the VS, the ANOVA revealed no significant main effect of group (F_(1, 62)_= 3.48, *p*= 0.07, *η*_*p*_^*2*^= 0.05), but a main effect of condition (F_(1, 62)_= 88.63, *p* < 0.001, *η*_*p*_^*2*^= 0.59). Higher PSC was found during the anticipation of monetary reward as compared to the verbal condition (M_monetary_= 0.09 ± 0.15; M_verbal_= -0.04 ± 0.11). In addition, there was a group by condition interaction (F_(1, 62)_= 6.05, *p*= 0.02, *η*_*p*_^*2*^= 0.09), driven by a larger difference between monetary and verbal condition in the OCD than in the TD group (OCD: M_monetary_= 0.13 ± 0.16; M_verbal_= -0.03 ± 0.13; TD: M_monetary_= 0.05 ± 0.11; M_verbal_= -0.05 ± 0.09; t_(62)_= 2.46, *p*= 0.02, d= 0.62, for details see Fig. 2). No further main or interaction effects occurred in the VS (all *p*s > 0.33). Using SES as a covariate did not change our main finding of the group by condition interaction for the VS (see S13 for details). In addition, adding age and sex as further covariates did not change the interaction effect. For the OFC, data revealed no main or interactions effects (all* p*s > 0.37).

#### Feedback phase – ROI analysis

The ANOVA for the VS revealed no main effect of group (F_(1, 62)_ = 0.13, *p*= 0.72, *η*_*p*_^*2*^= 0.002) but main effects of condition (F_(1, 62)_ = 25.96, *p* < 0.001, *η*_*p*_^*2*^ = 0.30) and feedback (F_(1, 62)_= 61.77, *p* < 0.001, *η*_*p*_^*2*^ = 0.50). The main effect of condition was driven by higher PSC for the monetary relative to the verbal condition (M_monetary_ = -0.18 ± 0.24; M_verbal_ = -0.07 ± 0.20). The main effect of feedback indicated less negative PSC for the feedback ‘fast enough’ compared to ‘too slow’ (M_fast_enough_ = -0.08 ± 0.20; M_too_slow_ = -0.18 ± 0.21). Further, a condition by feedback interaction effect occurred (F_(1, 62)_ = 41.46, *p* < 0.001, *η*_*p*_^*2*^ = 0.40), indicating less negative PSC during the verbal condition when participants received the feedback *fast enough* (Feedback ‘fast enough’: M_monetary_ = -0.11 ± 0.23; M_verbal_ = -0.05 ± 0.21; Feedback ‘too slow’: M_monetary_ = -0.25 ± 0.26; M_verbal_ = -0.10 ± 0.20). There were no further interaction effects (all *p*s > 0.14).

For the OFC, a main effect of feedback emerged (F_(1, 62)_ = 13.45, *p* < .001, *η*_*p*_^*2*^ = .178) with higher PSC when receiving the feedback ‘fast enough’ (M_fast_enough_ = 0.046±0.26; M_too_slow_ = 0.009 ± 0.235). In addition, there was a condition by feedback interaction effect (F_(1, 62)_ = 11.352, *p* < .001, *η*_*p*_^*2*^ = .155) with the highest PSC during the feedback ‘fast enough’ in the monetary condition (Feedback ‘fast enough’: M_monetary_ = 0.06 ± 0.26; M_verbal_ = 0.03±0.26; Feedback ‘too slow’: M_monetary_ = -0.01± 0.23; M_verbal_ = 0.025 ± 0.26). No further main or interaction effects occurred (all *p*s > 0.140). Using SES as a covariate did not change main results (see S13 for details).

#### Correlation between neural activation in ROIs and severity of OCD symptoms

Data revealed no correlations between PSC in VS and the severity of OCD measured by the CY-BOCS.

## Results - Session II: Loss

### Behavioral results – reaction times

The 2x2x3 ANOVA revealed no main effect of group (*F*_(1, 55)_= 2.93, *p*= .09, *η*_*p*_^*2*^= 0.05) but a main effect of condition (*F*_(1, 55)_= 58.79, *p* < .001, *η*_*p*_^*2*^= 0.52) with faster RTs in the monetary than the verbal condition (M_monetary_= 249.28 ± 25.80; M_verbal_ = 265.97±35.28). Further, a main effect of outcome probability (*F*_(1.216,110)_= 228.73, *p* < .001, *η*_*p*_^*2*^= *0.81)* with fastest RTs in the 67% outcome probability (M_12%_= 278.68 ± 36.88; M_34%_= 255.87 ± 29.12; M_67%_= 238.33 ± 25.59; t_12_34%_ (56)= 11.48, *p* < 0.001, d= 1.52; t_12_67%_(56)= 16.97, *p* < 0.001, d= 2.25; t_34_67%_(56)= 18.84, *p* < 0.001, d= 2.50) occurred (for details see S8 and S7 with SES as covariate)*.* No interactions were found (all *ps* > 0.37).

### fMRI results

#### Anticipation phase – ROI analysis

The ANOVA for the VS revealed no main effect of group (F_(1, 55)_= 3.90, *p*= 0.05, *η*_*p*_^*2*^= 0.07). However, there was a main effect of condition (F_(1, 55)_= 98.24, p < 0.001, *η*_*p*_^*2*^= 0.64) driven by higher PSC during the anticipation of monetary loss compared to verbal condition (M_monetary_= 0.05 ± 0.22; M_verbal_= -0.12 ± 0.21), and a group by condition interaction occurred (F_(1, 55)_= 5.25, *p* = 0.026, *η*_*p*_^*2*^= .087). The groups differed in terms of the differential activation between monetary and verbal condition (OCD: M_monetary_= 0.13 ± 0.23; M_verbal_= -0.09 ± 0.24; TD: M_monetary_= -0.02 ± 0.19; M_verbal_= -0.15 ± 0.18; see Fig. 2). Also, there was a main effect of probability (F_(2, 110)_= 3.47, *p*= 0.04, *η*_*p*_^*2*^= 0.06) resulting from less negative PSC in 34% probability (M_12%_= -0.06 ± 0.23; M_34%_= -0.03 ± 0.20, M_67%_= -0.03 ± 0.21; t_12_34%_ (56)= -2.64, *p* = 0.01, d= -0.35; t_12_67%_ (56) = -1.91, *p*= 0.06, d= -0.25; t_34_67%_(56)= 0.42, *p* = 0.34, d= 0.06). No further interaction effects occurred (all other *ps* > 0.46). Using SES as a covariate did not change our main finding of the interaction between group and condition for the VS (see S14). Adding age and sex as further covariates did not change the interaction effect.

Regarding OFC, data only revealed a main effect of condition (F_(1, 55)_= 9.47, *p*= 0.003, *η*_*p*_^*2*^= 0.15) with less negative PSC during the verbal condition (M_monetary_= -0.04 ± 0.21; M_verbal_= -0.01± 0.18; all other *p*s > 0.32).

#### Feedback phase - ROI analysis

Focusing on the VS, data revealed no main effect of group (F_(1**, **55)_ = 0.997,* p* = 0.32, *η*_*p*_^*2*^ = 0.02) but a main effect of condition (F_(1**, **55)_= 58.37, *p* < 0.001, *η*_*p*_^*2*^ = 0.52) and a main effect of feedback (F_(1**, **55)_= 41.16, *p* < 0.001, *η*_*p*_^*2*^= 0.43). The main effect of condition was driven by less negative PSC in the VS for the verbal condition (M_monetary_= -0.43 ± 0.35; M_verbal_= -0.26 ± 0.31) while the effect of feedback indicated less negative PSC for the feedback ‘fast enough’ compared to the feedback ‘too slow’ (M_fast_enough_= -0.29 ± 0.34; M_too_slow_= -0.40 ± 0.31). No interactions were found (all other *p*s > 0.07).

Regarding the OFC a main effect of feedback (F_(1, 55)_=8.03, *p*= 0.01, *η*_*p*_^*2*^= 0.127) occurred. The feedback ‘fast enough’ yielded to positive PSC whereas ‘too slow’ to negative PSC (M_fast_enough_= 0.01 ± 0.36; M_too_slow_= -0.03 ± 0.36). Using SES as a covariate did not change the results (see S14).

#### Correlation between neural activation in ROIs and severity of OCD symptoms

There were no correlations between ROI activation and symptom severity during the anticipation or feedback phase for loss.

## Discussion

Based on previous findings of altered reward processing in adult patients with OCD [46], the current fMRI study examined neural activation in I) reward and II) loss anticipation and receipt comparing adolescents with OCD and TD controls during a MID task with varying outcome probabilities. We focused specifically on the VS and OFC which are implicated in reward and loss processing and both are also associated with neural deviations in OCD [25, 47].

In line with previous developmental research conducted in adolescents and adults including patient populations [18, 40, 48, 49], the MID task used here recruited bilateral VS during both sessions, but with greater VS activation in adolescents with OCD than TD during the anticipation phase. In contrast, research in adults with OCD findings ranged from no differences to hypoactivation of the VS when compared to controls [2, 18, 50]. Further, aberrant activation of OFC has been found in adults with OCD [47, 51], but in our study with adolescents with OCD only during the feedback phase of the loss session when controlling for socioeconomic status. When comparing previous studies in adults with OCD with our findings in adolescents, it is important to consider developmental processes since parts of the reward circuit mature during adolescence until adulthood [52].

Contrasting brain activation of typical adolescents and adults, previous studies using the MID task found hypoactivation of the VS in adolescents compared to adults during the anticipation phase [26, 27, 53–55]. Interestingly, here we demonstrated hyperactivation of the VS during the anticipation of reward and loss in adolescents with OCD. One could speculate that the nature of the MID task with its unchanging stimulus-response-outcome associations (i.e., the constant relationship between cue color, reaction, and outcome probability) may be more motivating to adolescents with OCD than to TD but not necessarily more rewarding given the lack of activation differences in the feedback phase. Parts of the ventral-affective circuit, like the VS, are of importance for both habit formation and expression, but also in terms of goal-directed behavior [56]. Thus, in OCD simple reactions that resemble habits or serve the purpose of avoiding harm (i.e., not receiving or losing a reward) might up-regulate VS activation when expecting upcoming rewards or losses. In the longer term, this may contribute to excessive habit formation as reflected in hyperactivation of the caudate nucleus among patients with OCD [7, 57]. This idea ties in well with current conceptualizations of compulsions in OCD [2, 58], which suggest that harm avoidance behavior is the core dimension of OCD [59]. However, the VS hyperactivation may also reflect a desire to avoid ‘outcome uncertainty’ more generally, which contributes to the impaired decision-making often observed in this patient group [14]. Additionally, at least in the current study, the neural response of adolescents with OCD appeared to be selective and directed towards valuable tangible resources, like money, as the OCD group presented a greater activation difference between monetary and verbal condition. However, follow-up brain imaging studies using different types of tangible (e.g., food) and non-tangible (e.g., praise/reprimands) stimuli are warranted to further support such a conclusion. Considering the developing brain, the current finding could indicate an even more accentuated reward system in adolescents with OCD, leading to a more pronounced imbalance. The divergence from previous studies conducted exclusively on adults with OCD may suggest that hyperactivation of the VS during anticipation of reward and loss is a neural feature of early-onset OCD, which would support the view discussed in the literature that it is a distinct subtype of OCD [24]. However, the discrepancies between our results and those of previous studies of adults with OCD could also simply be related to the age of the participants at the time the study was conducted. To disentangle the confounding effect between the subtypes of OCD and the age of study participants, future research is needed that directly compares early-onset (i.e., diagnosed in childhood or adolescence) and late-onset OCD (i.e., beginning in adulthood) and follows the course of OCD in longitudinal studies.

At the neural level, the MID task allows an anticipation phase to be disentangled from a feedback phase when processing reward and loss. While the former is associated with the motivational drive to obtain a favorable outcome (‘wanting’), the latter refers to the actual hedonic response to obtaining the outcome (‘liking’; [60]). As OCD often begins in adolescence, the hyperactivation of the VS during the anticipation phase of both reward and loss may suggest that adolescent OCD is more closely linked to the ‘wanting’ of a reward as well as the desire to prevent a potential loss. Moreover, the VS is involved in habit formation, and OCD has been associated with a reliance on the habit system leading to compulsions [61]. Given the changing VS circuitry during adolescence [62], early intervention appears to be of paramount importance as it offers great potential for treatment success. The lack of between-group differences in the OFC is consistent with developmental models of adolescence, such as the maturational imbalance model of Casey et al. [63], according to which prefrontal regions are still less mature in adolescence compared to the VS. While adults with OCD have most commonly been found to have atypical prefrontal activations, including the OFC [64], adolescents with OCD may be more strongly guided by aberrant ‘wanting’ responses in the VS. The lack of group differences in brain activation during the feedback phase suggests that adolescent OCD may be maintained less by aberrant ‘liking’ responses. However, further studies directly comparing adolescent with adult OCD are needed to substantiate such an explanation.

VS activation also depends on the probability with which a reward or loss is estimated [26, 65, 66] and is linked to cognitive effort through mobilizing cognitive resources in correspondence to varying task demands [67, 68]. However, this varies with age as previously demonstrated [26]. For example, age differences in VS activation for high (i.e. 66%, 88%) but not for a low reward probability of 33% (i.e. highest task demands) were found when comparing typical adolescents with typical adults. Adolescents may therefore seem to experience behaviors as more rewarding that include greater mobilization of cognitive resources. However, this clear gradation at the neural level was not found in our study. This, in turn, could suggest that the groups do not differ in terms of their willingness to exert effort and the possible rewarding response they might get depending on different outcome probabilities. However, independent of outcome probability, it is arguable that, at least in part, the observed group differences could also be due to generally greater cognitive effort in adolescents with OCD as compared to TD adolescents. Finally, we did not find correlations between symptom severity (i.e. CY-BOCS score) and PSC in the VS or OFC during reward and loss. Current imaging results are therefore not directly related to OCD symptom severity. Our data do not support the assumption that adolescents with OCD lack the rewarding response that follows the execution of a behavior as indicated by absent group differences during the feedback phase. Instead, results suggest that adolescents with OCD are more motivated to gain or retain a valuable resource like money as indicated by group differences during the anticipation phase of both reward and loss.

In addition, behaviorally, participants reacted faster when anticipating a monetary compared to a verbal (control) outcome in both sessions. This confirms previous studies in various clinical and non-clinical populations [48, 69, 70]. We also did not find between-group differences regarding overall RTs, which is in line with previous work [18].

In summary, we found a stronger activation difference between the monetary and verbal condition in the VS during the anticipation phase, but not during the feedback phase, for both potential rewards and losses in adolescents with OCD compared to TD adolescents. Thus, adolescents with OCD appear to be more responsive to the monetary condition (whether reward or loss). Part of the current data can be interpreted in line with the concept of harm avoidance (i.e. not losing), as adolescents with OCD were found to have higher PSC during the anticipation of a possible loss of money. However, adolescents with OCD seem not only to be more motivated and prepared to avoid a potential monetary loss but also to gain a reward. This might offer new insights into the influence of the reward system on OCD symptoms.

### Limitations and further directions

The present study compared monetary and verbal trials. Therefore, the verbal condition might have been perceived as relatively negative in the reward session due to the absent possibility of gaining a (tangible) reward and vice versa in the loss session. Diverging results from other studies may be linked to differences in task designs [71]. Furthermore, subsequent studies that integrate reward and loss trials within the same task could find out more about the relation of these two processes in OCD. It is important to note that early-onset OCD is usually more common in boys than girls, especially in childhood [72], but the current study sample included more girls than boys with OCD. This may have influenced our findings and therefore, sex-specific effects related to reward and loss processing need to be further investigated in larger, better-powered studies. While the focus of this work was to gain initial insight into neural reward and loss processing of adolescents with OCD, it is important to note that the current sample was selected to avoid confounding factors related to comorbidities that are common in adolescent OCD. For example, we excluded patients with co-occurring ADHD because previous work has shown altered reward processing in this patient group (e.g., [69]). Although the current approach likely limits the generalizability of the current findings to the entire population of adolescents with OCD, they do provide insight into the pathophysiology that may be specific to adolescent OCD. However, future research should investigate the interplay of reward and loss processing in OCD and the influence of comorbidities. Last, we cannot completely rule out that adolescents with OCD were more motivated by the prospect of getting money than TD, especially against the background of differences in socioeconomic status. Although including socioeconomic status as a covariate did not change the main results, caution is necessary when addressing differences in socioeconomic status within the framework of an ANCOVA [73]. Questionnaires that measure subjective motivation to earn money as well as individual value of money might further provide insights and should be included in following studies.

## Conclusions

The results of the current study support the hypothesis of altered anticipation of (monetary) reward and loss in adolescents with OCD. These findings are particularly important for understanding abnormal reward and loss processing in adolescent compared to adult OCD. We note that Shephard et al. [15] have developed a profile describing changes within the ventral-affective circuitry associated with OCD etiology, but this profile is primarily based on studies of adults with OCD. It attempts to explain not only the decreased sensitivity to reward, but also the increased anticipation of punishment (e.g., losses). However, our study of adolescents with OCD revealed a somewhat different picture, indicating increased sensitivity to both reward and loss in the VS. This suggests that compulsive behaviors that begin in childhood or adolescence may become entrenched over time and that the underlying mechanisms may change or adapt over the course of the illness or manifest somewhat differently in late-onset OCD in adulthood. In sum, our findings fill in an important gap in (adolescent) OCD research and offer an important first insight into the early-onset of the disorder and its association with key regions of the ventral-affective circuit. Future research focusing on the developmental aspects of OCD is necessary to inform new, better tailored and thus more effective treatments for adolescents and adequate timing of therapeutic interventions before the disorder is fully developed.

### Supplementary Information

Supplementary Material 1.

## Data Availability

Data are available from the corresponding authors on reasonable request.

## Abbreviations

ADHD: Attention deficit hyperactivity disorder
BOLD: Blood-oxygen level dependent (response)
CSTC: Cortico-striatal-thalamo-cortical (circuit)
CY-BOCS: Children’s Yale-Brown Obsessive-Compulsive Scale
ERP: Exposure and response prevention (therapy)
fMRI: Functional magnetic resonance imaging
ICD-10: International Statistical Classification of Diseases and Related Health Problems, Version 10
IGT: Iowa gambling task
ITI: Intertrial interval
MID: Monetary incentive delay (task)
MINI: Mini-international neuropsychiatric interview
MRI: Magnetic resonance imaging
OC: Obsessive-compulsive (symptoms)
OCD: Obsessive-compulsive disorder
OCI: Obsessive-Compulsive Inventory-Revised
OFC: Orbitofrontal cortex
ROI: Region-of-interest
RT: Reaction time
SOA: Stimulus onset asynchrony
TD: Typically developing (controls)
VS: Ventral striatum
ZWIK: Zwangsinventar für Kinder und Jugendliche
