# Supplementary material for: Altered neural anticipation of reward and loss but not receipt in adolescents with obsessive-compulsive disorder
Source: BMC Psychiatry. 2024 May 14;24:362. doi: 10.1186/s12888-024-05808-x (PMC11094903; doi:10.1186/s12888-024-05808-x)
Supplement: Supplementary file 1 — Supplementary Material 1. [file 12888_2024_5808_MOESM1_ESM.docx]

**Supplementary Material**

**S1** Sample characteristics for the reward and loss session and exclusion after first scan appointment

|  |  | Reward session | | Loss session | |
| --- | --- | --- | --- | --- | --- |
|  |  | OCD | TD | OCD | TD |
| Diagnosis |  | 3,2 % |  | 3,8 % |  |
|  |  |  |  |  |  |
|  | Predominantly compulsive acts *[obsessional rituals]* |  |  |  |  |
|  | Mixed obsessional thoughts and acts | 96,8% |  | 96,2 % |  |
| Comorbidities subordinate to OCD | |  |  |  |  |
|  | Adjustment disorder | 2 |  | 1 |  |
|  | mild depressive episode | 1 |  | - |  |
|  | phobic anxiety disorder of childhood | 1 |  | - |  |
|  | Trichotillomania | 1 |  | 1 |  |
| Medication: | |  |  |  |  |
| Naïve | | 15 |  | 12 |  |
| Previously | | 4 |  | 3 |  |
| Currently with: | |  |  |  |  |
|  | Fluoxetine | 10 |  | 8 |  |
|  | Fluvoxamine | 1 |  | 1 |  |
|  | Sertraline | 1 |  | 2 |  |
|  |  |  |  |  |  |
|  | |  |  |  |  |
|  |  |  |  |  |  |
|  |  |  |  |  |  |
|  |  |  |  |  |  |
|  |  |  |  |  |  |
|  |  |  |  |  |  |
|  | |  |  |  |  |

**S2** Example of a calculation of the outcome probabilities used within the MID task.

The maximum reaction time (RT) window for each outcome probability varied in the beginning of the paradigm. The first percentage indicates the probability for reward, the second for loss. Each probability started with a different valid RT window:

88%/12% outcome probability − 400 ms

66%/34% outcome probability −300 ms

33%/67% outcome probability − 200 ms

Dependent on the outcome probability, participants had to react within the valid RT window. For example, they had to react within 400ms for the valid RT window of the 88%/12% outcome probability. Afterwards, the RT window adapted in response to the individual RT depending on a positive (i.e. successful - reacted within valid RT window, ‘fast enough’) or negative (i.e. unsuccessful – missed the valid RT window, ‘too slow’) feedback.

Successful: RTwindow = RTwindow − (RTwindow * 0.03)

Unsuccessful: RTwindow = RTwindow + (RTwindow * 0.07)

The last numbers were inverse for the 33%/67% outcome probability and 0.05 each for the 66%/34% outcome probability. Note that for all three outcome probabilities, a lower limit of 100 ms and an upper limit of 1000 ms was defined as the valid RT window. We used adaptive RT windows to keep hit (resp. error) rates constant between participants and outcome probability conditions. This approach was inspired by other studies in the field of reward and punishment processing that used customized RT windows (e.g., van Dongen et al. 2015, *Mol Autism*).

**S3** Raking for the different outcome probabilities in the reward and loss session.

|  | OCD | | TD | |  |  |  |  |
| --- | --- | --- | --- | --- | --- | --- | --- | --- |
|  | *Mean Rank* | *Sum of Ranks* | *Mean Rank* | *Sum of Ranks* |  | *U-test Statistic* | Standardized test statistics | *p* |
| Reward |  |  |  |  |  |  |  |  |
| Yellow 33% | 30.94 | 959.00 | 33.03 | 1057.00 |  | 463.00 | -1.32 | 0.19 |
| Blue 66% | 29.71 | 921.00 | 34.22 | 1095.00 |  | 425.00 | -1.07 | 0.28 |
| Pink 88% | 34.90 | 1082.00 | 20.19 | 934.00 |  | 463.00 | -0.50 | 0.62 |
| Loss |  |  |  |  |  |  |  |  |
| Pink 12% | 26.19 | 681.00 | 28.71 | 804.00 |  | 330.00 | -0.63 | 0.53 |
| Blue 34% | 30.23 | 786.00 | 24.96 | 699.00 |  | 293.00 | -1.33 | 0.19 |
| Yellow 67% | 26.58 | 691.00 | 28.36 | 794.00 |  | 340.00 | -.44 | 0.66 |

*Note.* In the reward session one participant was not able to rank the outcome probabilities and in the loss session three participants were not able to rank. For the ranking, participants ratings were converted to numbers 1-3 with 1 indicating the lowest win and 3 the highest. Higher mean ranks therefore indicate that participants rated the probability higher in terms of reward or loss.

| S4 Error rates for OCD and TD group in the reward and loss session. | | | | | | | | | | | | | |  |  |
| --- | --- | --- | --- | --- | --- | --- | --- | --- | --- | --- | --- | --- | --- | --- | --- |
|  | | OCD | | TD | | Post-Hoc Tests - Comparison between groups | | | | | | | | | |
|  | | *Reward*  *(n=31)* | *Loss  (n= 26)* | *Reward*  *(n= 33)* | *Loss*  *(n= 31)* | *Reward* | | | | | *Loss* | | | | |
|  | | *M (SD)* | *M (SD)* | *M (SD)* | *M (SD)* | *Mean Difference* | *t* | *df* | *p_bonf_* | *d* | *Mean Difference* | *t* | *df* | *p_bonf_* | *d* |
| Total | | 1.90 *(1.16)* | 2.08 *(1.14)* | 1.98 *(1.40)* | 1.82 *(1.06)* | 0.08 | 0.25 | 62 | 0.80 | .06 | 0.27 | 0.91 | 55 | 0.37 | .24 |
| Monetary condition | | 2.07 *(1.27)* | 2.45 *(1.66)* | 2.21 *(1.66)* | 2.02 *(1.31)* | 0.15 | 0.40 | 62 | 0.69 | .10 | 0.43 | 1.10 | 55 | 0.28 | .29 |
| Verbal condition | | 1.73 *(1.31)* | 1.72 *(0.96)* | 1.75 *(1.34)* | 1.61 *(1.13)* | 0.02 | 0.05 | 62 | 0.96 | .01 | 0.11 | 0.38 | 55 | 0.71 | .10 |
| **Outcome Probability 33/67%** | | | | |  |  |  |  |  |  |  |  |  |  |  |
| Total | | 1.86 *(1.16)* | 2.42 *(1.32)* | 2.09 *(1.81)* | 2.16 *(1.24)* | 0.24 | 0.62 | 62 | 0.54 | .15 | 0.26 | 0.77 | 55 | 0.44 | .21 |
| Monetary condition | | 2.03 *(1.60)* | 2.89 *(2.07)* | 2.61 *(2.18)* | 2.68 *(1.62)* | 0.57 | 1.19 | 62 | 0.24 | .30 | 0.21 | 0.42 | 55 | 0.67 | .11 |
| Verbal condition | | 1.68 *(1.50)* | 1.96 *(1.19)* | 1.58 *(1.84)* | 1.65 *(1.40)* | 0.10 | 0.24 | 62 | 0.81 | .06 | 0.32 | 0.91 | 55 | 0.37 | .24 |
| **Outcome Probability 66/34%** | | | | |  |  |  |  |  |  |  |  |  |  |  |
| Total | | 1.92 *(1.48)* | 2.06 *(1.40)* | 1.92 *(1.57)* | 1.79 *(1.36)* | 0.01 | 0.01 | 62 | 0.99 | .003 | 0.27 | 0.73 | 55 | 0.47 | .19 |
| Monetary condition | | 1.87 *(1.48)* | 2.04 *(1.66)* | 1.94 *(1.82)* | 1.61 *(1.50)* | 0.07 | 0.17 | 62 | 0.87 | .04 | 0.43 | 1.02 | 55 | 0.31 | .27 |
| Verbal condition | | 1.97 *(1.91)* | 2.08 *(1.70)* | 1.91 *(1.77)* | 1.97 *(1.56)* | 0.06 | 0.13 | 62 | 0.90 | .03 | 0.11 | 0.25 | 55 | 0.80 | .07 |
| **Outcome Probability 88/12%** | | |  |  |  |  |  |  |  |  |  |  |  |  |  |
| Total | | 1.92 *(1.50)* | 1.77 *(1.38)* | 1.92 *(1.51)* | 1.50 *(1.14)* | 0.01 | 0.01 | 62 | 0.99 | .003 | 0.27 | .81 | 55 | 0.42 | .22 |
| Monetary condition | | 2.29 *(1.76)* | 2.42 *(2.19)* | 2.09 *(2.10)* | 1.77 *(1.59)* | 0.20 | 0.41 | 62 | 0.68 | 0.10 | 0.65 | 1.29 | 55 | 0.20 | .34 |
| Verbal condition | | 1.55 *(1.65)* | 1.12 *(1.18)* | 1.76 *(1.60)* | 1.23 *(1.31)* | 0.21 | 0.52 | 62 | 0.61 | -0.13 | -0.11 | -0.33 | 55 | 0.74 | -.09 |
|  | *Note. d:* Effect size (Cohens’d), where 0.2 is small, 0.5 is medium and > 0.8 is large. The depicted mean values refer to number of trials.  **S5** Statistical analysis whole brain for reward as well as loss session.  Both phases – anticipation and feedback – were analyzed separately for both sessions (i.e., reward and loss).  Anticipation phase - The first-level analysis included six regressors of interest:   1. factor condition (monetary vs. verbal) 2. factor outcome probability (33% reward /67% loss vs. 66% reward /34% loss vs. 88% reward /12% loss)   Feedback phase - The first-level analysis included four regressors of interest:   1. factor condition (monetary vs. verbal) 2. factor feedback (fast enough vs. too slow)   The factors in both phases were modeled as stick functions at the point of cue presentation including the duration of the event and convolved with a canonical hemodynamic response function. Additionally, the onsets for error trials (i.e. trials, in which the participants did not react after the flash or values outside of the individually calculated range of mean ± 2 *SD*s), target and keypress and six movement regressors that resulted from rigid body realignment were included in the model as regressors of no interest. A minimum cluster size of 10 voxel was set. All brain coordinates are reported in MNI atlas space and are FWE-corrected using the FWE correction preselection threshold of *p* < 0.05. | | | | | | | | | | | | | | |

**S6** Reaction times for both groups (n = 64) in the reward session.

|  | OCD (*n = 31*) |  | TD (*n = 33*) |  | Post-Hoc Tests - Comparison between groups | | | | |
| --- | --- | --- | --- | --- | --- | --- | --- | --- | --- |
|  | *M (SD) in ms* |  | *M (SD) in ms* |  | *Mean Difference* | *t* | *df* | *P_bonf_* | *d* |
| Total | 245.70 (31.30) |  | 258.48 (28.14) |  | 13.44 | -1.81 | 62 | 0.08 | -0.45 |
| Monetary condition | 238.46 (*29.95*) |  | 250.48 (*26.22*) |  | 12.03 | -1.71 | 62 | 0.09 | -0.43 |
| Verbal condition | 251.63 (*34.08*)  *t*(30) = -5.19; *p* < 0.001 |  | 266.48 (*31.19*)  *t*(32) = -7.41; *p* < 0.001 |  | 14.85 | -1.82 | 62 | 0.07 | -0.46 |
| **Outcome Probability 33 %** |  |  |  |  |  |  |  |  |  |
| Total | 229.57 (*26.38*) |  | 238.51 (*21.11*) |  | 8.94 | -1.50 | 62 | 0.14 | -0.38 |
| Monetary condition | 223.32 (*25.44*) |  | 230.36 (*19.48*) |  | 7.04 | -1.25 | 62 | 0.22 | -0.31 |
| Verbal condition | 235.83 (*28.17*)  *t*(30) = 7.05; *p* < 0.001 |  | 246.66 (*23.99*)  *t*(32) = 8.31; *p* < 0.001 |  | 10.84 | -1.66 | 62 | 0.10 | -0.42 |
| **Outcome Probability 66 %** |  |  |  |  |  |  |  |  |  |
| Total | 244.21 (*30.46*) |  | 256.87 (*27.21*) |  | 12.66 | -1.76 | 62 | 0.08 | -0.44 |
| Monetary condition | 237.39 (*30.12*) |  | 250.75 (*27.17*) |  | 13.36 | -1.87 | 62 | 0.07 | -0.47 |
| Verbal condition | 251.04 (*32.79*)  *t*(30) = 4.79; *p* < 0.001 |  | 262.99 (*29.09*)  *t*(32) = 4.88; *p* < 0.001 |  | 11.95 | -1.55 | 62 | 0.13 | -0.39 |
| **Outcome Probability 88 %** |  |  |  |  |  |  |  |  |  |
| Total | 261.34 (*38.76*) |  | 280.050 *(38.72)* |  | 18.72 | -1.93 | 62 | 0.06 | -0.48 |
| Monetary condition | 254.66 (*36.55*) |  | 270.33 (*36.48*) |  | -15.67 | -1.72 | 62 | 0.09 | -0.43 |
| Verbal condition | 268.01 (*44.65*)  *t*(30) = 2.92; *p* = 0.007 |  | 289.72 *(43.32)*  *t*(32) = -5.46; *p* < 0.001 |  | 21.76 | -1.98 | 62 | 0.05 | -0.50 |

*Note.* *d*: Effect size (Cohens’d), where 0.2 is small, 0.5 is medium and > 0.8 is large

**S7** Behavioral results with socioeconomic status (Winkler index) as covariate in the reward (n = 64) and loss session (n = 57).

**Reward session**

The ANCOVA revealed no main effect of group (*F*_(1, 62)_= 2.20, *p* = 0.14, *η_p_^2^*= *0.35).* There was no main effect of condition (*F*_(1, 62)_= 0.46, *p* = 0.50, *η_p_^2^*= *0.01)* but of outcome probability (*F*_(1.212,122)_= 4.01, *p* = 0.04, *η_p_^2^= 0.06).* Participants demonstrated fastest RTs at 33% and slowest RTs at 88% outcome probability (M_33%_= 234.06, SEM=2.99; M_66%_= 250.58, SEM=3.61; M_88%_=270.74, SEM= 4.85). No interactions were found (all *ps* > 0.14).

**Loss session**

The ANCOVA demonstrated neither a main effect of group (*F*_(1, 62)_= 1.40, *p* = 0.24, *η_p_^2^*= *0.03)* nor of condition (*F*_(1, 62)_= 0.51, *p* = 0.48, *η_p_^2^*= *0.01)* but a main effect of outcome probability (*F*_(1.216,108)_= 5.09, *p* = 0.02, *η_p_^2^*= *0.09)* occurred. Participants were fastest at 33% and demonstrated slowest RTs at 88% outcome probability (M_12%_= 278.21, SEM= 4.84; M_34%_= 255.42, SEM= 3.81; M_67%_=237.92, SEM= 3.36). Data revealed no interactions (all *ps* > 0.17).

**S8** Reaction times for both groups (*n = 57*) in the loss session.

|  | OCD *(n* = 26) | TD *(n* = 31) | Post-hoc Tests – Comparisons between groups | | | | | | | | | | | | |  |  |
| --- | --- | --- | --- | --- | --- | --- | --- | --- | --- | --- | --- | --- | --- | --- | --- | --- | --- |
|  | *M (SD) in ms* | *M (SD)* in ms | *Mean Difference* | *t* | | *df* | | | *p_bonf_* | | | | *d* | | |  |  |
| Total | 250.37 *(28.71)* | 263.72 *(29.78)* | -13.35 | -1.71 | | 55 | | | 0.09 | | | | -0.46 | | |  |  |
| Monetary condition | 244.30 *(24.95)* | 253.46 *(26.17)* | -9.15 | -1.34 | | 55 | | | 0.19 | | | | -0.36 | | |  |  |
| Verbal condition | 256.44 *(33.81)* | 273.97 *(35.02)* | -17.54 | -1.91 | | 55 | | | 0.06 | | | | -0.51 | | |  |  |
|  | *t(*25) = -4.05; *p* < 0.001 | *t(*30) = 6.89; *p* < 0.001 |  |  |  | |  | | |  | | | |  | | |  |
| Outcome probability 12 % | 270.39 *(35.80)* | 285.64 *(36.91)* | -15.25 | -1.58 | | 55 | | | 0.12 | | | | -0.42 | | |  |  |
| Monetary condition | 265.26 *(32.99)* | 274.52 *(31.83*) | -9.26 | -1.08 | | 55 | | | 0.29 | | | | -0.29 | | |  |  |
| Verbal condition | 275.52 *(44.45)* | 296.77 *(45.82)* | -21.25 | -1.77 | | 55 | | | 0.08 | | | | -0.47 | | |  |  |
|  | *t(*25) = -1.65; *p* = 0.11 | *t(*30) = -4.44; *p* < 0.001 |  |  |  | | |  | | | |  | | |  | |  |
| Outcome probability 34 % | 248.64 *(28.34)* | 261.93 *(28.82)* | -13.29 | -1.75 | | 55 | | | 0.09 | | | | -0.47 | | |  |  |
| Monetary condition | 243.29 *(24.78)* | 252.08 *(25.85)* | -8.78 | -1.30 | | 55 | | | 0.20 | | | | -0.35 | | |  |  |
| Verbal condition | 253.99 *(32.92)* | 271.78 *(33.42)* | -17.79 | -2.02 | | 55 | | | 0.05 | | | | -0.54 | | |  |  |
|  | *t(*25) = -4.03; *p* < 0.001 | *t(*30) = -6.97; *p* < 0.001 |  |  |  | | |  | | | |  | | |  | |  |
| Outcome probability 67 % | 232.08 *(24.64)* | 243.58 *(25.57)* | -11.49 | -1.72 | | 55 | | | 0.09 | | | | -0.46 | | |  |  |
| Monetary condition | 224.36 *(21.33)* | 233.78 *(23.30)* | -9.42 | -1.58 | | 55 | | | 0.12 | | | | -0.42 | | |  |  |
| Verbal condition | 239.80 *(28.57)* | 253.37 *(29.25)* | -13.57 | -1.76 | | 55 | | | 0.08 | | | | -0.47 | | | | |
|  | *t(*25) = -7.38; *p* < 0.001 | *t(*30) = -8.08; *p* < 0.001 |  |  |  | | |  | | | |  | | |  | |  |
| *Note.* *d*: Effect size (Cohens’d), where 0.2 is small, 0.5 is medium and > 0.8 is large | | | | | | | | | | | | | | | |  |  |
| **S9** Functional activation in the reward session during the anticipation and feedback phase (n = 64), k > 10.   \|  \| L/R \|  \| Peak-voxel (mm) \| \| \|  \| *F/t*-value \| FWE corrected  *p*-value \| Cluster size *k* \| \| --- \| --- \| --- \| --- \| --- \| --- \| --- \| --- \| --- \| --- \| \| Brain region \|  \|  \| x \| y \| z \|  \|  \|  \|  \| \| **Anticipation phase** \|  \|  \|  \|  \|  \|  \|  \|  \|  \| \| **Main effect of group**  No activation on a whole brain level \|  \|  \|  \|  \|  \|  \|  \|  \|  \| \| **monetary > verbal condition** \|  \|  \|  \|  \|  \|  \|  \|  \|  \| \| VS \| L \|  \| -8 \| 10 \| 0 \|  \| 7.99 \| < 0.001 \| 225 \| \|  \| R \|  \| 20 \| 12 \| -8 \|  \| 6.83 \| < 0.001 \| 250 \| \| Lingual gyrus \| R \|  \| 12 \| -88 \| -6 \|  \| 7.84 \| < 0.001 \| 1982 \| \| Fusiform Gyrus \| R \|  \| 38 \| -50 \| -18 \|  \| 6.84 \| < 0.001 \| 80 \| \| Supplementary motor cortex \| R \|  \| 4 \| 4 \| 54 \|  \| 6.73 \| < 0.001 \| 198 \| \|  \| L \|  \| -4 \| 6 \| 50 \|  \| 6.42 \| < 0.001 \| 169 \| \| Precentral gyrus \| L \|  \| -50 \| 2 \| 42 \|  \| 6.46 \| < 0.001 \| 61 \| \|  \| R \|  \| 42 \| 6 \| 28 \|  \| 6.14 \| < 0.001 \| 81 \| \| Superior parietal lobule \| L \|  \| -26 \| -72 \| 32 \|  \| 6.36 \| < 0.001 \| 105 \| \|  \| R \|  \| 20 \| -66 \| 50 \|  \| 5.77 \| 0.001 \| 57 \| \| Middle occipital gyrus \| R \|  \| 38 \| -74 \| 20 \|  \| 5.92 \| < 0.001 \| 125 \| \| Inferior occipital gyrus \| L \|  \| -44 \| -84 \| -6 \|  \| 5.50 \| < 0.001 \| 25 \| \| Middle frontal gyrus \| L \|  \| -28 \| 0 \| 52 \|  \| 5.86 \| < 0.001 \| 41 \| \|  \| R \|  \| 34 \| 42 \| 32 \|  \| 5.67 \| < 0.001 \| 43 \| \| Anterior insula \| R \|  \| 28 \| 20 \| -10 \|  \| 5.40 \| 0.002 \| 11 \| \| Cerebellum \| R \|  \| 4 \| -64 \| -14 \|  \| 5.26 \| 0.002 \| 11 \| \| **verbal > monetary condition** \|  \|  \|  \|  \|  \|  \|  \|  \|  \| \| Angular gyrus \| L \|  \| -48 \| -72 \| 36 \|  \| 5.11 \| < 0.001 \| 19 \| \| **Main effect of probability** \|  \|  \|  \|  \|  \|  \|  \|  \|  \| \| Occipital pole \| R \|  \| 14 \| -96 \| 2 \|  \| 25.90 \| < 0.001 \| 161 \| \| **Feedback phase** \|  \|  \|  \|  \|  \|  \|  \|  \|  \| \| **monetary > verbal condition** \|  \|  \|  \|  \|  \|  \|  \|  \|  \| \| Superior frontal gyrus \| R \|  \| 22 \| 58 \| 26 \|  \| 5.46 \| 0.001 \| 17 \| \| **verbal > monetary condition** \|  \|  \|  \|  \|  \|  \|  \|  \|  \| \| Angular gyrus \| L \|  \| -28 \| -72 \| 48 \|  \| 10.54 \| < 0.001 \| 11709 \| \|  \| R \|  \| 34 \| -64 \| 46 \|  \| 10.15 \|  \| Part of same cluster \| \| Middle temporal gyrus \| L \|  \| -56 \| -46 \| -12 \|  \| 10.13 \| < 0.001 \| 915 \| \| Superior temporal gyrus \| R \|  \| 68 \| -22 \| 8 \|  \| 5.78 \| < 0.001 \| 22 \| \|  \| L \|  \| -56 \| -6 \| -2 \|  \| 5.20 \| 0.002 \| 13 \| \| Middle frontal gyrus \| R \|  \| 30 \| 16 \| 58 \|  \| 8.84 \| < 0.001 \| 747 \| \|  \| L \|  \| -50 \| 30 \| 30 \|  \| 7.52 \| < 0.001 \| 847 \| \| Superior frontal gyrus \| L \|  \| -2 \| 54 \| -4 \|  \| 8.81 \| < 0.001 \| 910 \| \|  \| R \|  \| 4 \| 44 \| -6 \|  \| 7.82 \|  \| Part of same cluster \| \| VS \| L \|  \| -8 \| 12 \| -4 \|  \| 8.72 \| < 0.001 \| 329 \| \|  \| R \|  \| 8 \| 14 \| -4 \|  \| 8.08 \| < 0.001 \| 413 \| \| Superior frontal gyrus \| L \|  \| -24 \| 24 \| 56 \|  \| 8.71 \| < 0.001 \| 934 \| \| Posterior cingulate gyrus \| L \|  \| 0 \| -30 \| 38 \|  \| 8.61 \| < 0.001 \| 600 \| \| Caudate \| R \|  \| 18 \| 4 \| 24 \|  \| 7.45 \| < 0.001 \| 244 \| \| Anterior orbital gyrus \| R \|  \| 24 \| 38 \| -6 \|  \| 6.35 \| < 0.001 \| 178 \| \| Medial orbital gyrus \| L \|  \| -26 \| 38 \| -18 \|  \| 6.23 \| < 0.001 \| 68 \| \| Precentral gyrus \| L \|  \| -20 \| -24 \| 56 \|  \| 6.09 \| < 0.001 \| 34 \| \|  \| R \|  \| 40 \| -12 \| 40 \|  \| 5.20 \| 0.003 \| 11 \| \| Occipital pole \| L \|  \| -22 \| -100 \| 4 \|  \| 7.44 \| < 0.001 \| 1160 \| \| Cerebellum \| L \|  \| -42 \| -72 \| -28 \|  \| 5.69 \| < 0.001 \| 41 \| \| **Fast enough > too slow** \|  \|  \|  \|  \|  \|  \|  \|  \|  \| \| VS \| R \|  \| 20 \| 12 \| -10 \|  \| 10.20 \| < 0.001 \| 840 \| \|  \| L \|  \| -16 \| 6 \| -10 \|  \| 10.11 \| < 0.001 \| 793 \| \| Precentral gyrus \| L \|  \| -24 \| -26 \| 66 \|  \| 7.50 \| < 0.001 \| 4426 \| \|  \| R \|  \| 44 \| -4 \| 52 \|  \| 7.05 \| < 0.001 \| 526 \| \| Middle frontal gyrus \| L \|  \| -36 \| 46 \| 22 \|  \| 6.50 \| < 0.001 \| 265 \| \|  \| R \|  \| 34 \| 46 \| 30 \|  \| 5.41 \| < 0.001 \| 35 \| \| Supramarginal gyrus \| R \|  \| 64 \| -38 \| 36 \|  \| 6.17 \| < 0.001 \| 178 \| \|  \| L \|  \| -64 \| -30 \| 26 \|  \| 5.89 \| < 0.001 \| 206 \| \| Middle temporal gyrus \| L \|  \| -52 \| -62 \| 8 \|  \| 5.49 \| < 0.001 \| 50 \| \| Operculum \| R \|  \| 44 \| -32 \| 22 \|  \| 5.60 \| < 0.001 \| 23 \| \| Anterior insula \| L \|  \| -34 \| 0 \| 2 \|  \| 5.48 \| 0.002 \| 12 \| \| Superior occipital gyrus \| L \|  \| -18 \| -88 \| 24 \|  \| 5.48 \| < 0.001 \| 23 \| \|  \| R \|  \| 24 \| -90 \| 26 \|  \| 5.13 \| 0.002 \| 12 \| \| Occipital pole \| R \|  \| 12 \| -96 \| 18 \|  \| 5.29 \| 0.002 \| 12 \| \| **Too slow > fast enough** \|  \|  \|  \|  \|  \|  \|  \|  \|  \| \| No activation on a whole brain level \|  \|  \|  \|  \|  \|  \|  \|  \|  \| \| **Interaction condition x feedback** \|  \|  \|  \|  \|  \|  \|  \|  \|  \| \| Occipital pole \| R \|  \| 20 \| -92 \| -6 \|  \| 67.83 \| < 0.001 \| 1961 \| \| Inferior occipital gyrus \| R \|  \| 40 \| -88 \| -6 \|  \| 59.90 \|  \| Part of same cluster \| \| Calcarine cortex \| R \|  \| 12 \| -92 \| 0 \|  \| 54.45 \|  \| Part of same cluster \| \|  \| L \|  \| -14 \| -92 \| -2 \|  \| 55.28 \| < 0.001 \| 1377 \| \| Posterior cingulate gyrus \| L \|  \| -6 \| -52 \| 24 \|  \| 59.38 \| < 0.001 \| 1659 \| \| Precuneus \| R \|  \| 14 \| -62 \| 26 \|  \| 56.79 \|  \| Part of same cluster \| \| Angular gyrus \| R \|  \| 32 \| -66 \| 38 \|  \| 58.10 \| < 0.001 \|  \| \| Middle occipital gyrus \| R \|  \| 46 \| -70 \| 32 \|  \| 50.14 \|  \| Part of same cluster \| \| Middle temporal gyrus \| R \|  \| 60 \| -48 \| -12 \|  \| 49.52 \| < 0.001 \| 194 \| \| Inferior temporal gyrus \| R \|  \| 52 \| -58 \| -14 \|  \| 32.50 \|  \| Part of same cluster \| \| Superior frontal gyrus medial segment \| L \|  \| -10 \| 54 \| -6 \|  \| 44.11 \| < 0.001 \| 186 \| \| Anterior cingulate gyrus \| L \|  \| -2 \| 46 \| -2 \|  \| 36.48 \|  \| Part of same cluster \| \| Angular gyrus \| L \|  \| -26 \| -76 \| 42 \|  \| 40.10 \| < 0.001 \| 358 \| \| Superior frontal gyrus medial segment \| R \|  \| 8 \| 56 \| 0 \|  \| 36.81 \| < 0.001 \| 22 \| \| Cerebellum exterior \| L \|  \| -36 \| -74 \| -28 \|  \| 36.66 \| < 0.001 \| 26 \| \| Supramarginal gyrus \| R \|  \| 48 \| -36 \| 52 \|  \| 35.08 \| < 0.001 \| 50 \| \| Putamen \| R \|  \| 18 \| 14 \| 0 \|  \| 29.58 \| < 0.001 \| 30 \| | | | | | | | | | | |  |  |  |  |  |  |  |

Note.: There were no further main effects or interactions on a whole brain level.

**S10** Functional activation in the reward session. Both groups are contrasted during the anticipation phase of the MID task when anticipating the monetary vs. the verbal (control) condition. For illustration purposes of the whole brain results, the preselection threshold was set to *p* < 0.001 uncorrected with a minimum cluster size of 10 voxels. Monetary and verbal (control) condition are contrasted for each group separately.


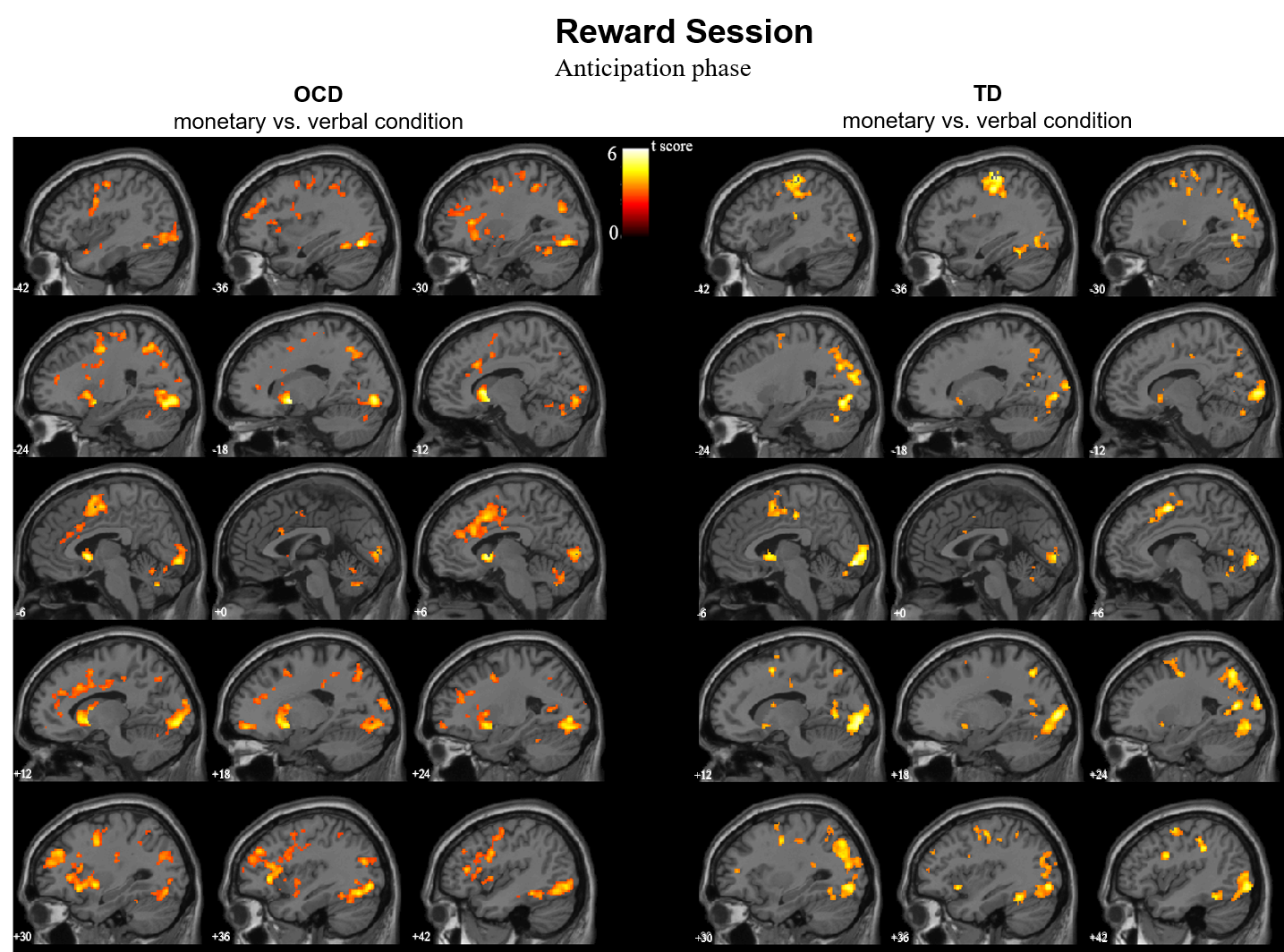


**S11** Functional activation in the loss session during the anticipation and feedback phase (n = 57), k > 10.

|  | L/R |  | Peak-voxel (mm) | | |  | *F/t*-value | FWE corrected  *p*-value | Cluster size *k* |
| --- | --- | --- | --- | --- | --- | --- | --- | --- | --- |
| Brain region |  |  | x | y | z |  |  |  |  |
| **Anticipation phase** |  |  |  |  |  |  |  |  |  |
| **Main effect of group**  No main effect on a whole brain level |  |  |  |  |  |  |  |  |  |
| **monetary > verbal condition** |  |  |  |  |  |  |  |  |  |
| VS | L |  | -8 | 10 | 0 |  | 6.32 | < 0.001 | 37 |
|  | R |  | 8 | 8 | 0 |  | 6.00 | < 0.001 | 16 |
| Precentral gyrus | L |  | -34 | -24 | 58 |  | 5.41 | < 0.001 | 54 |
| Anterior insula | R |  | 34 | 18 | -6 |  | 5.23 | 0.003 | 10 |
| Cerebellum | R |  | 4 | -60 | -14 |  | 5.21 | 0.001 | 15 |
|  |  |  |  |  |  |  |  |  |  |
| **verbal > monetary condition** |  |  |  |  |  |  |  |  |  |
| No activation on a whole brain level |  |  |  |  |  |  |  |  |  |
|  |  |  |  |  |  |  |  |  |  |
| **Feedback phase** |  |  |  |  |  |  |  |  |  |
| **OCD > TD** |  |  |  |  |  |  |  |  |  |
| Cerebellum exterior | L |  | -26 | -62 | -14 |  | 5.68 | 0.001 | 22 |
| Angular gyrus | L |  | -46 | -54 | 18 |  | 5.34 | 0.002 | 14 |
| **OCD < TD** |  |  |  |  |  |  |  |  |  |
| No activation on a whole brain level |  |  |  |  |  |  |  |  |  |
|  |  |  |  |  |  |  |  |  |  |
| **monetary > verbal condition** |  |  |  |  |  |  |  |  |  |
| Superior frontal gyrus | R |  | 8 | 22 | 64 |  | 6.66 | < 0.001 | 289 |
| Supplementary motor cortex | R |  | 8 | 22 | 34 |  | 6.16 |  | Part of same cluster |
| Anterior insula | R |  | 30 | 22 | -8 |  | 6.66 | < 0.001 | 189 |
|  | L |  | -34 | 18 | -10 |  | 6.27 | < 0.001 | 80 |
| Lingual gyrus | R |  | 16 | -92 | -6 |  | 6.09 | < 0.001 | 229 |
| Calcarine cortex | L |  | -8 | -92 | -2 |  | 5.62 |  | Part of same cluster |
| Middle temporal gyrus | R |  | 50 | -30 | -4 |  | 5.89 | < 0.001 | 25 |
| Supramarginal gyrus | R |  | 46 | -46 | 54 |  | 5.78 | < 0.001 | 110 |
| Superior parietal lobule | R |  | 30 | -64 | 34 |  | 5.73 | < 0.001 | 49 |
| Middle frontal gyrus | R |  | 44 | 8 | 34 |  | 5.31 | 0.004 | 10 |
| **verbal > monetary condition** |  |  |  |  |  |  |  |  |  |
| Orbital gyrus | L |  | -24 | 28 | -14 |  | 7.91 | < 0.001 | 332 |
| Middle frontal gyrus | L |  | -30 | 26 | 54 |  | 7.67 | < 0.001 | 311 |
| Caudate | R |  | 20 | -6 | 26 |  | 7.50 | < 0.001 | 119 |
|  | L |  | -10 | 12 | -6 |  | 7.03 | < 0.001 | 90 |
| Anterior cingulate gyrus | L |  | -6 | 42 | -8 |  | 7.13 | < 0.001 | 794 |
| Medial frontal cortex | R |  | 2 | 38 | -16 |  | 6.98 |  | Part of same cluster |
|  | L |  | -4 | 32 | -16 |  | 6.67 |  | Part of same cluster |
| Precuneus | L |  | -6 | -56 | 16 |  | 6.71 | < 0.001 | 144 |
| Angular gyrus | L |  | -44 | -76 | 40 |  | 6.32 | < 0.001 | 204 |
|  |  |  |  |  |  |  |  |  |  |
| **Fast enough > too slow** |  |  |  |  |  |  |  |  |  |
| VS | L |  | -10 | 10 | -4 |  | 10.19 | < 0.001 | 525 |
|  | R |  | 10 | 12 | 2 |  | 9.09 | < 0.001 | 583 |
| Supplementary motor cortex | R |  | 4 | 6 | 52 |  | 7.61 | < 0.001 | 5039 |
| Postcentral gyrus | R |  | 16 | -30 | 68 |  | 7.29 |  | Part of same cluster |
| Superior occipital gyrus | R |  | 20 | -88 | 20 |  | 6.45 | < 0.001 | 200 |
|  | L |  | -16 | -92 | 22 |  | 5.78 | < 0.001 | 38 |
| Posterior insula | L |  | -34 | -14 | -4 |  | 6.36 | < 0.001 | 63 |
| Middle temporal gyrus | R |  | 40 | -58 | 12 |  | 6.15 | < 0.001 | 55 |
|  | L |  | -46 | -68 | 10 |  | 5.98 | < 0.001 | 136 |
| Transverse temporal gyrus | L |  | -44 | -26 | 10 |  | 5.51 | 0.001 | 16 |
| Middle occipital gyrus | L |  | -42 | -74 | 16 |  | 5.09 |  | Part of same cluster |
| Lingual gyrus | L |  | -10 | -76 | -10 |  | 6.12 | < 0.001 | 63 |
| Putamen | R |  | 32 | -16 | -2 |  | 6.12 | 0.001 | 17 |
| Angular gyrus | R |  | 66 | -50 | 18 |  | 5.84 | 0.001 | 23 |
| Supramarginal gyrus | L |  | -64 | -32 | 30 |  | 5.82 | < 0.001 | 138 |
|  | R |  | 66 | -36 | 32 |  | 5.35 | < 0.001 | 28 |
| Central operculum | R |  | 50 | 4 | 2 |  | 5.74 | < 0.001 | 88 |
| Middle frontal gyrus | L |  | -36 | 40 | 38 |  | 5.70 | < 0.001 | 111 |
| Cerebellum exterior | R |  | 46 | -54 | -34 |  | 5.69 | 0.004 | 10 |
| Anterior insula | L |  | -28 | 28 | 0 |  | 5.66 | < 0.001 | 47 |
|  | R |  | 34 | 8 | 10 |  | 5.15 | 0.002 | 14 |
| Posterior insula | R |  | 32 | -24 | 12 |  | 5.42 | 0.002 | 14 |
| **Too slow > fast enough** |  |  |  |  |  |  |  |  |  |
| Anterior cingulate gyrus | L |  | 0 | 42 | 2 |  | 5.68 | < 0.001 | 33 |
| Angular gyrus | R |  | 38 | -62 | 50 |  | 5.61 | < 0.001 | 38 |
| Occipital pole | R |  | 18 | -98 | -6 |  | 5.57 | 0.002 | 15 |
| Supramarginal gyrus | R |  | 50 | -44 | 50 |  | 5.13 | 0.002 | 13 |
| **Interaction condition x feedback** |  |  |  |  |  |  |  |  |  |
| Anterior cingulate gyrus | L |  | 0 | 40 | 2 |  | 38.40 | < 0.001 | 86 |
|  | R |  | 4 | 34 | 22 |  | 36.14 | < 0.001 | 41 |
| Inferior temporal gyrus | R |  | 52 | -54 | -12 |  | 36.30 | < 0.001 | 29 |
| Superior parietal lobule | R |  | 28 | -64 | 40 |  | 34.60 | < 0.001 | 36 |
| Anterior insula | R |  | 36 | 16 | -6 |  | 31.95 | 0.001 | 15 |
| Posterior cingulate gyrus | R |  | 6 | -38 | 38 |  | 31.75 | 0.003 | 10 |
| Occipital pole | L |  | -20 | -104 | 4 |  | 31.33 | < 0.001 | 46 |
|  |  |  |  |  |  |  |  |  |  |

Note.: There were no further main effects or interactions on a whole brain level.

**
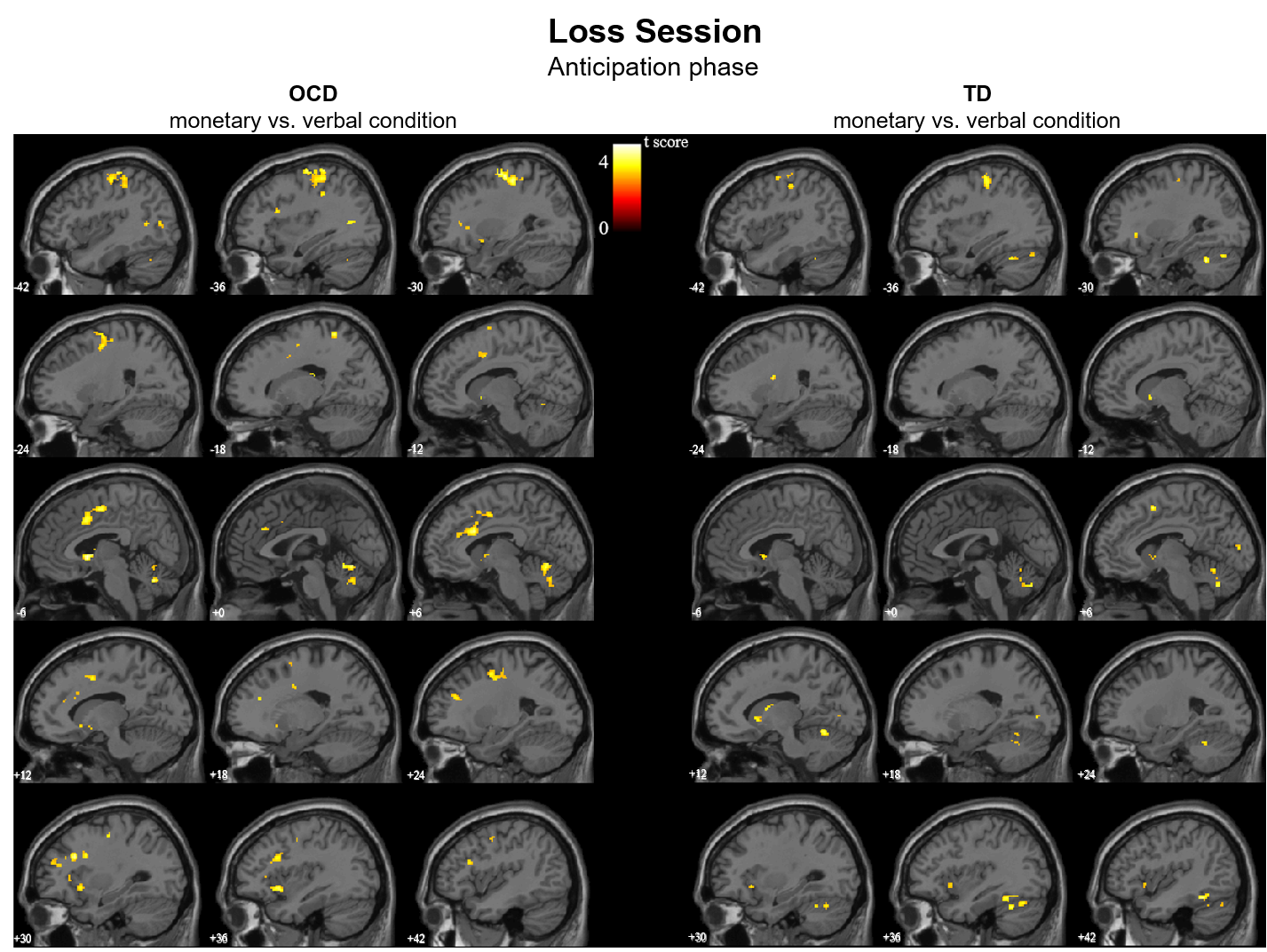
S12** Functional activation in the loss session. Both groups are contrasted during the anticipation phase of the MID task when anticipating the monetary vs. the verbal (control) condition. For illustration purposes of the whole brain results, the preselection threshold was set to *p* < 0.001 uncorrected with a minimum cluster size of 10 voxels. Monetary and verbal (control) condition are contrasted for each group separately.

**S13** Results of ROI analyses including socioeconomic status (SES) as covariate during the anticipation and feedback phase in the reward session *(n* = 64).

Anticipation phase

For the VS, data demonstrated a main effect of group (F_(1, 61)_= 4.07, *p*= 0.048, *η_p_^2^*= 0.06) with positive PSC in the OCD group in contrast to negative PSC in the TD group (M_OCD_= 0.05, SEM= 0.02; M_TD_= -0.01, SEM= 0.02). Moreover, there was no main effect of condition (F_(1, 61)_= 0.57, p = 0.45, *η_p_^2^*= 0.01) but a group by condition interaction occurred (F_(1, 61)_= 7.71, *p*= 0.01, *η_p_^2^*= .11) resulting from differences in activation between monetary and verbal condition between the groups (OCD: M_monetary_= 0.14, SEM= 0.25; M_verbal_= -0.03, SEM= 0.21; TD: M_monetary_= 0.039, SEM= 0.03; M_verbal_= -0.05, SEM= 0.02). Also, there was a three-way interaction between condition x probability x SES (F_(2, 122)_= 3.12, *p*= 0.048, *η_p_^2^*= 0.05). No further main effects or interactions occurred (all other *ps* > 0.05).

For the OFC, data only revealed a trend towards a three-way interaction condition x probability x SES (F_(1.707, 122)_= 3.27, *p*= 0.05, *η_p_^2^*= 0.05). No main effects or interactions occurred (all *p*s > 0.08).

Feedback phase

In the VS, only a trend towards an interaction condition x group occurred (F_(1, 61)_ = 0.13, *p* = 0.076, *η_p_^2^*= 0.051; all other *p*s > 0.12).

Regarding the OFC, no main effects or interactions occurred (all *p*s > 0.15).

**S14** Results of ROI analyses including socioeconomic status (SES) as covariate during the anticipation and feedback phase in the loss session *(n* = 57).

Anticipation phase

In the VS, the ANCOVA demonstrated a main effect of group (F_(1, 54)_ = 4.83, *p* = 0.03, *η_p_^2^*= 0.08) with generally positive PSC in the OCD group while the TD group demonstrated negative PSC (M_OCD_= 0.33_,_ SEM= 0.41; M_TD_= -0.10, SEM= 0.38). Further, a group x condition interaction emerged (F_(1, 54)_ = 5.80, *p* = 0.02, *η_p_^2^*= .097) driven by differences for the groups in activation between monetary and verbal condition (OCD: M_monetary_= 0.14_,_ SEM= 0.43; M_verbal_= -0.08, SEM= 0.44; TD: M_monetary_= -0.03, SEM= 0.04; M_verbal_= -0.16, SEM= 0.04). However, no further main effects or interactions were detected (all other *ps* > 0.18).

Regarding OFC, data only revealed only a trend towards a main effect of group (F_(1, 54)_ = 3.19, *p* = 0.08, *η_p_^2^*= 0.06; all other *p*s > 0.43).

Feedback Phase

#### For the VS, a trend towards an interaction between condition x group occurred (F_(1, 54)_ = 4.00, *p*= 0.05, *η_p_^2^*= 0.07) but there were no further main effects or interactions (all *p*s > 0.15).

#### For the OFC, there was a main effect of group (F_(1, 54)_ = 8.63, *p* = 0.01, *η_p_^2^* = 0.14) with positive PSC in the OCD group in contrast to negative PSC in the TD group when generally receiving a feedback (M_OCD_= 0.14_,_ SEM= 0.68; M_TD_= -0.14, SEM= 0.61). No other effects emerged (*p*s > 0.26).
